# Vaccination and Risk Aversion: Evidence From a Flu Vaccination Campaign

**DOI:** 10.1002/hec.70037

**Published:** 2025-09-24

**Authors:** Clémentine Garrouste, Arthur Juet, Anne‐Laure Samson

**Affiliations:** ^1^ LEDa‐LEGOS Université Paris Dauphine Paris France; ^2^ Academic Unit of Health Economics Faculty of Medicine and Health Leeds Institute of Health Sciences University of Leeds Leeds UK; ^3^ LEMMA University of Paris‐Panthéon Assas Paris France

**Keywords:** influenza, regression discontinuity design, risk aversion, vaccination campaign

## Abstract

We examine the causal effect of a French flu vaccination campaign on vaccination behavior. Individuals aged 65 and over receive an invitation letter with a voucher for a free flu shot, while those who are not eligible have to cover the costs themselves. Using a Fuzzy Regression Discontinuity Design, we find that receiving the invitation letter with the voucher leads to a significant increase in the likelihood of getting vaccinated. This effect is driven by individuals who are risk‐averse. As illustrated in our theoretical model, for them, the costs of influenza infection outweigh the costs of the vaccine's side effects.

## 1 Introduction

Viruses spread through social interactions. The consequences of their spread are costly for the health sector and, in turn, for society at large. Policies limiting interpersonal contacts (e.g., lockdowns) reduce disease prevalence. However, the reduction of social life may be harmful, especially for the elderly. Vaccination—if both available and effective—is the best way to control viral diseases without affecting social interactions. Therefore, a key economic focus is the individual behavioral response to policy decisions, such as vaccination campaigns.

In this paper, we focus on the seasonal influenza vaccination campaign in France, a country well‐known for its population's high level of mistrust in vaccination (Larson et al. 2015). The response to the influenza vaccination policy is a major concern in an aging population where the prevalence of respiratory diseases is increasing. In France, seasonal influenza affects an average of two to six million individuals per year (between 3% and 9% of the French population). Although the flu is generally mild, it can cause severe complications, sometimes fatal ones (HAS, 2023). All age groups are affected by seasonal influenza, but some high‐risk individuals are more vulnerable and can experience serious complications including very young children, 65 and older individuals, pregnant women, and individuals with chronic diseases (heart disease, renal failure, diabetes, respiratory failure) or immunosuppression (HIV, chemotherapy, etc.). On average, seasonal vaccination leads each year to about 1 million consultations with GPs, more than 20,000 hospitalizations and around 9000 deaths. More than 90% of the deaths occur among individuals aged 65 and over (Thompson et al. 2009). These figures highlight the significant impact of seasonal influenza on public health and public health expenditures. Simple hygiene measures can help limit person‐to‐person transmission. However, vaccination remains the best way to protect individuals against the flu: the risk of infection by the virus is reduced by 50% and the severity of the disease and the incidence of complications and deaths are reduced (WHO 2017). Vaccination is cost‐effective (Ting et al. 2017; White 2021), and the World Health Organization (WHO) recommends vaccinating 75% of individuals aged 65 and over each year (WHO 2019). Therefore, since 2000, France has implemented a national vaccination campaign for high‐risk individuals. Each year, chronically ill individuals and those aged 65 and above receive a voucher for a free flu shot, accompanied by a leaflet informing them about the dangers of influenza and the benefits of vaccination. Individuals not targeted by the program can also get vaccinated, but they have to pay for it and do not receive any invitation letters.

Using data from the 2014 Health and Social Protection Survey (ESPS), collected by the French Institute for Research and Documentation in Health Economics, we focus on the 2013/2014 French influenza vaccination campaign. Adopting a fuzzy regression discontinuity design around the 65 years old threshold, we assess the effect of this flu vaccination program in France on vaccination behavior. We also investigate the heterogeneity of the impact according to risk aversion.

We show that individuals who report receiving the invitation letter and who are therefore aware of their eligibility for free vaccination are 22–25 pp (percentage points) more likely to get vaccinated against the flu. This effect is driven by risk‐averse individuals, reflecting the smaller proportion of risk‐takers among compliers. Referring to theoretical mechanisms, this means that for them, the costs of influenza infection outweigh the costs of the vaccine's side effects, and the vaccination campaign lowered the level of risk aversion from which individuals get vaccinated.

Our paper adds to the existing literature in several ways. First, the paper contributes to the literature on the effectiveness of vaccination campaigns. Several recent papers show that vaccination campaigns (both information and mandatory campaigns) are effective at raising vaccination rates for the disease targeted and for the targeted population (Abrevaya and Mulligan 2011; Chang 2016; Churchill 2021; J.‐L.J. Hirani 2021; Lawler 2017, 2019; Ward 2014). Vaccination campaigns may also have unintended spillover effects on other vaccines (Carpenter and Lawler 2019; Churchill 2021; Garrouste et al. 2023; J. Hirani and Wüst 2024) or on individuals not targeted by the campaign (Bouckaert et al. 2020; J.‐L.J. Hirani 2021; Humlum et al. 2024). In this paper, we confirm the effectiveness of the influenza vaccination campaign on vaccination against the flu. Moreover, the magnitude of the estimated effect when we use the reduced form is comparable to the one found by Brilli et al. (2020) in Italy and Bouckaert et al. (2020) in the Netherlands, who also study an influenza vaccination campaign. Both papers study the impact of eligibility for the program on vaccination take‐up and are therefore in an “intention‐to‐treat” framework, which is highly informative. However, our data enable us to measure not only the impact of eligibility for the vaccination campaign but also the impact of awareness of that eligibility on vaccination behavior. We can observe whether individuals report receiving the voucher and the leaflet, which constitutes a contribution to the literature.

Second, we investigate the mechanisms that boost or undermine the effectiveness of the campaign and through which the awareness effect operates. Understanding these mechanisms is crucial to grasp the full spectrum of consequences associated with vaccination policies. We therefore add new insights to the literature by investigating the heterogeneous effects of vaccination campaigns according to preferences. The literature generally focuses on heterogeneous effects depending on gender (Brilli et al. 2020; Carpenter and Lawler 2019) or education and socio‐economic status (Brilli et al. 2020; J.‐L.J. Hirani 2021; Lawler 2017, 2019). In our case, we tested for heterogeneity in individual responses to the campaign according to risk aversion, which has received very little attention, despite the large literature that exists on the impact of risk aversion on preventive behavior. For example, being more risk‐averse has been shown to increase the uptake of medical tests (Goldzahl 2017; Picone et al. 2004) and decrease the probability of smoking, drinking, or being severely obese (Anderson and Mellor 2008). Understanding the heterogeneity according to risk aversion is crucial as the direction of the correlation between risk aversion and vaccination is unknown a priori. Both vaccination and non‐vaccination are risky. Vaccination results from a trade‐off between the risk of side effects due to vaccine injection and the risk of contracting the disease if not vaccinated (Courbage and Peter 2021). In the literature, Lepinteur et al. (2023); Nuscheler and Roeder (2016); Tsutsui et al. (2010) show that more risk‐averse individuals are more likely to get vaccinated, so infection is perceived as riskier than vaccine side effects. This is in line with our results. Crainich et al. (2019) also show theoretically that risk aversion increases the probability of being vaccinated when the disease is lethal. However, the empirical literature on this subject is quite limited.

Then, to better understand the mechanisms that explain the effectiveness of vaccination campaigns, we go further by characterizing those who are less reactive to the vaccination program. More precisely, we compare the characteristics of the compliers, that is, those who react positively to the campaign and get vaccinated due to the voucher and the information documentation, to those of the never‐takers, using the framework proposed by Marbach and Hangartner (2020). We find that compliers are more risk‐averse than never‐takers.

The paper proceeds as follows. Section 2 describes the French national influenza campaign and Section 3 the empirical strategy. Section 4 presents the data and discusses the main identification assumptions. Section 5 reports the main results as well as some robustness checks. Section 6 presents some theoretical mechanisms at stake. Finally, Section 7 discusses the results and concludes.

## 2 The 2013–2014 National Influenza Campaign

Influenza vaccination has been reimbursed by the French National Health Insurance (NHI) since 1985. In 1985, vaccination was offered free of charge to all individuals 75 years of age or older. In 1989, this age was reduced to 70 and to 65 in 2000 (Buisson et al. 2007). To follow the recommendations of the WHO and achieve the target of vaccinating 75% of individuals over 65 against seasonal influenza (WHO 2019), France has implemented a national vaccination campaign every autumn since 2000. This campaign focuses on individuals aged 65 and older, who are particularly vulnerable to serious complications, as well as another high‐risk group: those with chronic diseases.

**FIGURE 1:**
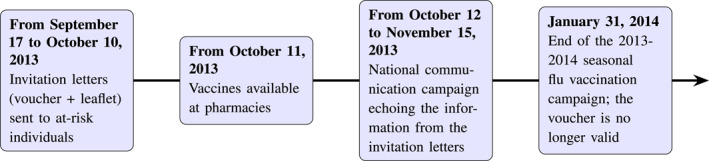
Timeline of the 2013–2014 national influenza campaign.

Our paper analyzes the 2013–2014 campaign, which was conducted in several stages from September 17, 2013 to January 31, 2014. The timeline of the campaign is illustrated in Figure 1 and additional details can be found in Section A of the Appendix.

First, from September 17 to October 10, 2013, all individuals aged 65 and over in 2013 as well as those who turned 65 during that calendar year (or who would turn 65), along with individuals with chronic diseases (i.e., at‐risk individuals identifiable through the NHI system) received a personalized invitation letter at home from the NHI. This letter included a vaccination voucher permitting free vaccination between October 11 and January 31, 2014 (see Supporting Information S1: Figure A1 in the appendix). Additionally, the letter contained a leaflet aimed at raising awareness among at‐risk individuals about the potential seriousness of influenza and the importance of vaccination as a safe and effective means of preventing the disease and its complications (see Supporting Information S1: Figure A2 in the appendix). The slogan was: “Flu is no joke. So I get vaccinated” (*“La grippe, ce n'est pas rien. Alors, je fais le vaccin”*).^1^ Other at‐risk individuals, such as pregnant women, individuals with morbid obesity (defined as a body mass index greater than 40 kg/$m^{2}$) and caregivers of babies under 6 months, could not be identified through the NHI system. Although they were also eligible for free vaccination, they did not receive invitations due to the lack of a comprehensive NHI screening. However, they could receive their vaccine at no cost between October 11 and January 31, 2014, during a consultation if they specifically requested it from their general practitioner (GP) or if the GP offered it. All other individuals not considered at risk were not eligible for free vaccination and had to pay for their flu shots. However, the NHI reimbursed 65% of the vaccine price and 70% of the GP consultation fee. During the 2013/2014 season, vaccine prices, established by various pharmaceutical companies, ranged from 5.36 euros to 6.25 euros, and a standard GP consultation cost 23 euros. For those ineligible for free vaccination, the out‐of‐pocket cost for the least expensive vaccine was approximately 1.90 euros and 6.90 euros for the consultation, totaling around 9 euros. This cost could be further reduced for individuals with complementary health insurance (85% of individuals in 2014), which typically reimbursed this out‐of‐pocket cost.

In the second step of the campaign, from October 12 to November 15, 2013, immediately following the distribution of the invitation letters, the NHI launched a communication campaign aimed at reminding at‐risk individuals of their receipt of the invitation letter. This campaign reminded them that they had received a voucher for free vaccination and disseminated the same information contained in the letter, namely the dangers of influenza and how to protect oneself through vaccination. Designed specifically for individuals over 65 and those with chronic diseases, the campaign featured radio and television spots showcasing these audiences. These ads aired multiple times a day on popular radio stations, with messages delivered by well‐known hosts while video spots were broadcast before televised programs available for replay. The campaign also included print media coverage, in targeted magazines (such as television and senior magazines), and regional newspapers. All individuals wishing to receive the vaccine could do so for free, from October 11 to January 31, 2014.

In parallel, during October and November, a communication campaign targeted healthcare professionals, including general practitioners, nurses, pharmacists, due to their advisory role and their ability to inform and reassure patients. They received an information letter from the NHI, accompanied by a practical note detailing available vaccines, the individuals eligible for 100% coverage, and the associated procedures, including coding and billing. Information materials, such as posters and videos, were also available to them.

In this paper, we aim to measure the causal effect of the campaign—specifically the impact of awareness regarding eligibility for free vaccination and the knowledge of risks associated with influenza and its complications—on vaccination behavior. We take advantage of the discontinuity in letter reception that occurs at the age of 65 to assess the causal effect of the campaign on vaccination behavior. Special attention will be paid to individuals with chronic diseases, the other group of individuals targeted by the campaign, particularly in the selection of the samples used for the econometric analysis.

## 3 Empirical Strategy

In order to empirically evaluate the causal impact of the influenza vaccination campaign on vaccination take‐up (V), we exploit the discontinuity in the reception of the invitation letter (R) at the age of 65, and use a regression discontinuity in a fuzzy design. More precisely, the local treatment effect τ is defined as:

(1) $$\tau =\frac{\lim_{a\to 65+}E\left(V|A=a\right)-\lim_{a\to 65-}E\left(V|A=a\right)}{\lim_{a\to 65+}E\left(R|A=a\right)-\lim_{a\to 65-}E\left(R|A=a\right)}$$

The variation in vaccination take‐up just before and after the 65 years old threshold is related to the variation in the proportion of individuals receiving the invitation letter (voucher and leaflet). An estimate τ is obtained using a 2SLS procedure that allows us to estimate a local average treatment effect (LATE) on the compliers, that is, on individuals who got vaccinated against the flu due to their awareness of eligibility for free vaccination and their knowledge of the risks associated with influenza, but who would not have chosen to get vaccinated otherwise.

The first stage measures the impact of the age threshold (65 yo) on reporting receiving the invitation letter at home $\left(R_{i}=1\right)$, that is, on awareness of eligibility for free vaccination and on the risks associated with the flu. This awareness is facilitated by the receipt of the leaflet and the voucher, with additional reinforcement provided by the communication campaign and the efforts of doctors, who reminded individuals of having received it and reiterated its content. Our specification is the following:

(2) $$R_{i}=\beta_{0}+\beta_{1}1_{A_{i}\geq 65}+\beta_{2}1_{A_{i}\geq 65}\times f\left(A_{i}-65\right)+\beta_{3}1_{A_{i}<65}\times g\left(A_{i}-65\right)+\nu_{i}$$

The second stage measures the impact of reporting receiving the invitation letter at home on vaccination take‐up. With $V_{i}$ equal to 1 if the individual i gets vaccinated against the flu and 0 otherwise, we estimate:

(3) $$V_{i}=\alpha_{0}+\alpha_{1}{\hat{R}}_{i}+\alpha_{2}1_{A_{i}\geq 65}\times f\left(A_{i}-65\right)+\alpha_{3}1_{A_{i}<65}\times g\left(A_{i}-65\right)+\varepsilon_{i}$$

Finally, the reduced‐form specification is:

(4) $$V_{i}=\gamma_{0}+\gamma_{1}1_{A_{i}\geq 65}+\gamma_{2}1_{A_{i}\geq 65}\times f\left(A_{i}-65\right)+\gamma_{3}1_{A_{i}<65}\times g\left(A_{i}-65\right)+u_{i}$$

The fuzzy‐regression discontinuity estimate, $\hat{\alpha_{1}}$ in Equation (3), is obtained as $\hat{\beta_{1}}/\hat{\gamma_{1}}$ from Equations (2) and (4). It measures the causal impact of the campaign on vaccination uptake that is, the impact of awareness regarding eligibility for free vaccination and the knowledge of risks associated with influenza and its complications.

$A_{i}$, the running variable, is the age in months, measured in December 2013 thanks to information on the month and year of birth of each individual. All individuals over 65 years of age in 2013 or who turned 65 during the calendar year 2013, should have received the voucher and the leaflet. $1_{A_{i}\geq 65}$ therefore denotes a dummy that defines eligibility for treatment. In statistical and econometric analysis, we restrict the sample so that $65-bw\leq A_{i}\leq 65+bw$, with bw being the bandwidth used around the threshold of 65. The choice of the bandwidth results from a bias/efficiency trade‐off: a smaller bandwidth decreases the bias while a larger bandwidth increases precision. Here we use the optimal bandwidth selection procedure of Calonico et al. (2014), which leads to a bandwidth of 44 months before and after the age of 65. Our sample therefore contains individuals aged between 61 years and 4 months and 68 years and 8 months in December 2013; see Table B3 in the Appendix. However, we performed robustness checks using larger or smaller bandwidths to test the sensitivity of our results to the choice of the bandwidth (see Section 5.3).

We pay special attention to modeling the underlying function of the running variable (Lee and Card 2008), by correctly specifying the functions f and g. We use a non‐parametric local linear specification with a triangular kernel function that assigns greater weight to observations near the threshold and a first‐degree polynomial in age, than can differ on each side of the cut‐off (Calonico et al. 2014). We present the coefficients using a conventional variance estimator. The bias‐corrected coefficients, along with both conventional and robust corrected standard errors, are presented in the Appendix, but the results are virtually the same (see Section 5.3). We also report estimates using parametric linear regressions to check the consistency of our results. In that case, $f\left(A_{i}-65\right)$ and $g\left(A_{i}-65\right)$ are simply defined as $\left(A_{i}-65\right)$ with different slopes before and after the threshold. Both methods are applied using a bandwidth of 44 months.

Our main estimates are obtained without the use of control variables, as they are continuously distributed around the threshold (see Section 4.4.1). However, to increase precision, we also run additional regressions including control variables, that is, gender, marital status, profession, education, and income.

Finally, we account for heterogeneous effects among individuals based on their level of risk aversion. Therefore, Equation (3) becomes:

(5) $$V_{i}=\delta_{0}+\delta_{1}RA_{i}+\delta_{2}{\hat{R}}_{i}+\delta_{3}\hat{R_{i}\times RA_{i}}+\delta_{4}1_{A_{i}\geq 65}\times f\left(A_{i}-65\right)+\delta_{5}1_{A_{i}<65}\times g\left(A_{i}-65\right)+\xi_{i}$$

where $RA_{i}=1$ if the individual declares being risk‐averse (see below for the construction of this variable) and ${\hat{R}}_{i}$ and $\hat{R_{i}\times RA_{i}}$ are obtained using two first‐stage regressions.^2^

Three conditions must be met to implement a fuzzy regression discontinuity design. First, the expectations of potential outcomes ($R_{i}$ and $V_{i}$), conditional on age, must be continuous at the age threshold. This condition cannot be tested directly. However, we verified that the variables related to the outcomes are continuously distributed at the age of 65 (see Section 4.4.1). Second, it is assumed that the LATE and treatment status are locally jointly independent of the individual's age. This implies that individuals cannot manipulate their age or misreport their age to the NHI to receive the invitation letter. We verified this assumption in Section 4.4.2 using the McCrary test for manipulation (McCrary (2008)). Third, we must assume that there are no ‘defiers,’ that is, individuals who would have been vaccinated in the absence of the campaign but chose not to be vaccinated because of the campaign.

## 4 Data

### 4.1 Health and Social Protection Survey

We use data from the 2014 wave of the Health and Social Protection Survey (ESPS), a survey conducted by the Institute for Research and Documentation in Health Economics, that provides a representative sample of the French population. We are focusing on the 2014 wave that includes detailed information on flu vaccination and invitation letter reception. However, it is important to note that 2014 is not a particular notable year in terms of influenza incidence; the preceding year, 2013, is also not an atypical year, as shown in Supporting Information S1: Figure B1.

In this survey, individuals were asked about the usual sociodemographic characteristics (age, sex, socioprofessional category, education, etc.). In addition, they were asked to report whether they received a voucher from the NHI in the autumn of 2013 for free flu vaccination. They were also asked if they had been vaccinated against the latest seasonal flu (see Supporting Information S1: Table B1 in the appendix). All individuals were interviewed between January 2014 and February 2015 (see Supporting Information S1: Table B2 in the appendix). Fifty percent of them were interviewed in February and March 2014, shortly after the end of the 2013/2014 vaccination campaign, allowing us to confidently state that their responses regarding vaccination pertain to the 2013/2014 campaign we are evaluating. Approximately 10% were interviewed in September, while another significant wave of interviews (30%) occurred between October and December 2014, coinciding with the 2014/2015 vaccination campaign. Respondents from this period may confuse their responses with regard to the *latest* seasonal flu. To address this potential source of confusion, we will conduct robustness checks (see Section 5.3.5).

The survey also contains information on the risk aversion of individuals (see Supporting Information S1: Table B1 in the appendix), measured using the Likert scale (0–10), as is common in the literature (Bonin et al. 2007; Dohmen et al. 2011; Dohmen et al. 2016; Jaeger et al. 2010; Lepinteur et al. 2023). According to Dohmen et al. (2011), this measure of risk aversion has proven reliable in predicting individual health behavior compared to the standard lottery measure. Massin et al. (2015) use the same measure to study vaccination behavior among general practitioners (GPs), who are a better informed population than the general population. They show that risk‐averse physicians receive more flu shots and recommend the vaccine more, which is in line with our results. In the survey, individuals are asked about their willingness to take risks: ‘When it comes to your attitude toward risk, where do you rank on a scale from 0 to 10′, where 0 means ‘not at all willing to take risks' and 10 means ‘very willing to take risks'. Note that this measure of risk aversion is not specifically related to health behavior but concerns all individual behaviors. We define as “risk‐averse” individuals who tend to avoid risk and answer values 0 to 4 to this question, and as “risk‐takers” individuals who answer 5 and more. However, Figure B2 in the Appendix shows a focal point with a value of ‘5′, which is typical for a measure of risk aversion (see, e.g., Lepinteur et al. (2023)). Consequently, in Section 5.3.4, we check the robustness of our results by including this focal point within the “risk‐averse” category and classifying people who report values of 5 and below as risk‐averse.

### 4.2 Samples Used for the Analysis

The initial database contains 15,729 individuals. We restrict the sample to those aged around 65 years old, using a bandwidth of 44 months, as detailed in Section 3. All individuals aged 65 and over in 2013 as well as those who turned 65 during that calendar year (or who will turn 65), are potentially affected by the campaign. To identify them in our sample, we use information on the month and year of birth and calculate their age in months as of December 2013. Individuals born in December 1948 or earlier are between 65 years old and 68 years and 8 months old in December 2013; they are defined as treated. Untreated individuals are those born in January 1949 or later, who are between 61 years and 4 months old and 64 years and 11 months old in December 2013 (see Supporting Information S1: Table B3 in the appendix). The final sample, which we will refer to as the *Whole Sample*, comprises 1698 individuals, including 882 untreated individuals and 857 treated individuals.

**FIGURE 2:**
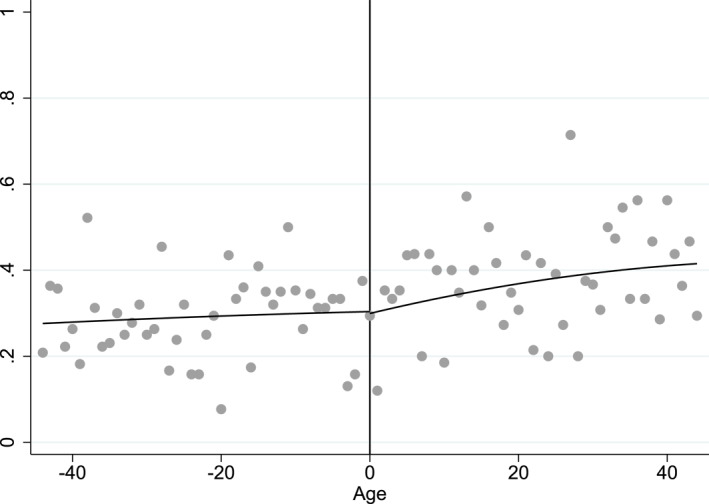
Percentage of individuals with a chronic disease by age (in months) on the whole sample ‐ zero cutoff for the 65 years old and bandwidth of 44 months. *Source:* ESPS 2014.

In this sample, individuals with chronic diseases are eligible for free vaccination regardless of their age.^3^ They receive the leaflet and the voucher, they are also concerned by the communication campaign and receive advice from their doctor whatever their age: they are not concerned by the age threshold and for them, we will see that there is no discontinuity at age 65. On average, in the sample, 29% of individuals aged between 61 and 64 have a chronic disease while 37% of individuals aged between 65 and 68 do. The proportion of chronically ill individuals may seem very high. However, it corresponds to the proportion observed in the French population. Using prevalence data of chronic diseases in France, by age, from the health insurance files, we obtain orders of magnitude that are very close to those observed in our data: 27% of individuals aged 60–64 years and 35% of those aged 65–69 years suffer from chronic disease. More details can be found in Section B6 in the Appendix. This difference is significant at the 5% level and can be attributed to the fact that older individuals are less likely to be in good health. However, the proportion of chronically ill individuals continuously varies at the 65‐year‐old threshold, which is confirmed in Figure 2.

In order to focus on individuals who are affected by the campaign solely due to their age, we construct a second sample, which we will refer to as the *Threshold‐based Sample*. All individuals with a chronic disease are excluded from this sample, regardless of whether they are under or over 65 years old. This sample comprises 1130 individuals, including 603 untreated individuals and 527 treated individuals.

### 4.3 Vaccination Related Outcomes

Table 1 presents descriptive statistics for the main variables used in the analysis for the Whole sample (top of the table) and the Threshold‐based sample (bottom of the table). Column 1 (resp. columns 2 and 3) displays the statistics for all individuals in the analyzed sample (resp. for the untreated individuals and for the treated individuals). Column 4 provides the coefficient and significance level from the test for equal means between treated and untreated individuals. On average, treated individuals in the whole sample are 64 pp more likely than untreated individuals to report receiving the flu vaccination letter, and this figure is 74 pp in the threshold‐based sample. As a consequence, 90% of people 65 and older report receiving the invitation letter; they are therefore aware of their eligibility for free vaccination and know the risks associated with influenza. Furthermore, in the whole sample (resp. in the threshold‐based sample), treated individuals are 15 pp more likely than untreated ones (resp. 17 pp) to get vaccinated against the flu.

**TABLE 1:** Comparison of treated and untreated individuals, using a bandwidth of 44 months around the 65 years old threshold, on the whole sample and the threshold‐based sample.

	(1)	(2)	(3)	(4)
	Sample	Non treated	Treated	
Whole sample	Mean	Mean	Mean	*T*‐test
**Outcomes**				
Flu invitation reception	0.58	0.27	0.91	0.64***
Flu vaccination take‐up	0.29	0.22	0.37	0.15***
**Socio‐demographic characteristics**				
*At the individual level:*				
In a relationship	0.84	0.83	0.84	0.00
Male	0.49	0.47	0.49	0.02
High school diploma	0.81	0.82	0.80	−0.02
Executive	0.23	0.23	0.23	0.01
Employee	0.34	0.33	0.35	0.01
Blue collar	0.43	0.43	0.42	−0.02
Pensioner	0.87	0.80	0.93	0.13***
With a chronic disease	0.33	0.29	0.37	0.07**
*At the household level:*				
Nb. of individuals	2.04	2.07	2.01	−0.06
Net income per unit of consumption	1949.56	1973.48	1936.20	−33.45
**Preferences**				
Risk‐averse	0.55	0.55	0.54	−0.01
Number of observations	1698	882	857	1698
**Threshold‐based sample**				
**Outcomes**				
Flu invitation reception	0.51	0.16	0.90	0.74***
Flu vaccination take‐up	0.22	0.14	0.31	0.17***
**Socio‐demographic characteristics**				
*At the individual level:*				
In a relationship	0.84	0.84	0.85	0.01
Male	0.45	0.44	0.46	0.02
High school diploma	0.84	0.85	0.82	−0.03
Executive	0.26	0.26	0.27	0.00
Employee	0.32	0.31	0.33	0.02
Blue collar	0.41	0.42	0.40	−0.02
Pensioner	0.86	0.79	0.93	0.14***
With a chronic disease	—	—	—	—
*At the household level:*				
Nb. of individuals	2.02	2.04	1.99	−0.05
Net income per unit of consumption	2063.78	2078.17	2047.10	−31.07
**Preferences**				
Risk‐averse	0.53	0.55	0.52	−0.03
Number of observations	1130	603	527	1130

*Note:* ***Statistically significant at the 1% level; ** at the 5% level; * at the 10% level. All figures are computed using a bandwidth of 44 months around the 65 years old threshold. Column (1) computes the mean for the entire sample, column (2) for the group of non‐treated individuals (below the age of 65), column (3) for the group of treated individuals (above the age of 65), and column (4) reports the coefficient and significance level of the test for equal means between the treated and untreated individuals. Figures at the top of the table are computed on the whole sample while figures at the bottom are computed on the threshold‐based sample, that is excluding individuals with chronic diseases, whatever their age.

*Source:* ESPS 2014.

**FIGURE 3:**
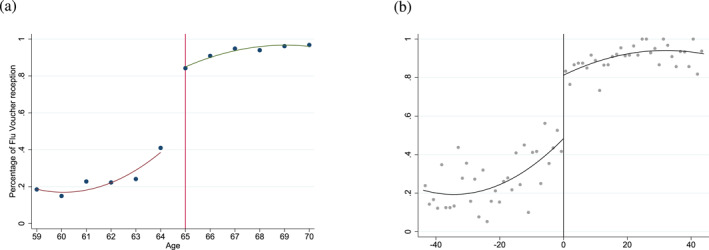
Flu vaccination invitation rate by age, on the whole sample. (a) Flu vaccination invitation rate by age (in years). (b) Flu vaccination invitation rate by age (in months) ‐ zero cutoff for the 65 years old and bandwidth of 44 months. *Source:* ESPS 2014.

**FIGURE 4:**
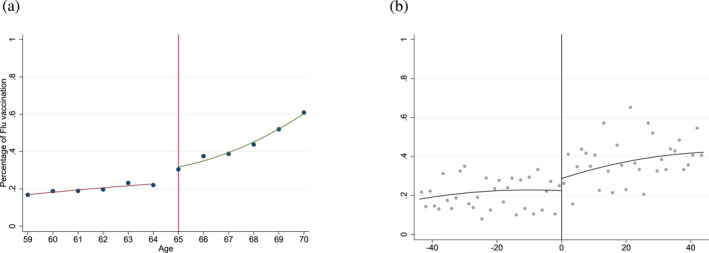
Flu vaccination rate by age, on the whole sample. (a) Flu vaccination rate, by age in years. (b) Flu vaccination rate by age in months ‐ zero cutoff for the 65 years old and bandwidth of 44 months. *Source:* ESPS 2014.

On the whole sample, decomposing these figures by age (Figure 3), we observe that the proportion of individuals who report receiving the letter at the age of 64 is approximately 40% and approximately 80% at the age of 65, an increase of 40 pp^4^. Note that the probability of reporting receiving the invitation does not range from 0% to 100%. In fact, some individuals over 65 years old may have received the invitation letter but did not remember it, discarded it without examining its content or there may have been postal issues, resulting in a proportion that does not reach 100% after the age threshold. Furthermore, individuals with chronic diseases are already eligible for free vaccination before the age of 65, which is why the proportion exceeds 0% prior to the age threshold. In the threshold‐based sample, where individuals with chronic diseases are excluded (on both sides of the threshold), the increase is more pronounced (see Supporting Information S1: Figure C1 in the appendix). Simultaneously, Figure 4 illustrates an increase, though less pronounced than the previous one, in the proportion of individuals vaccinated against the flu, rising from about 20% at age 64% to 30% at age 65. Once again, the probability of being vaccinated does not range from 0% to 100%: individuals not eligible for free vaccination may, however, opt for it by bearing the costs themselves (crossovers) and individuals eligible may not necessarily take it (imperfect compliance). In the threshold‐based sample, the increase is of the same magnitude, rising from 10% to 20% (see Supporting Information S1: Figure C1 in the appendix).

### 4.4 Identifying Assumptions

#### 4.4.1 Continuity in Individuals' Characteristics at the 65 years Old Threshold

In the empirical strategy, we use the discontinuity in the probability of receiving the invitation letter to measure the causal impact of the campaign on vaccination rates. To attribute the discontinuity in vaccination uptake around the threshold to the receipt of the invitation (voucher and leaflet), individuals below and above the 65‐year cut‐off should not systematically differ. If this condition is met, any discontinuous change in vaccination rates isolates the average causal impact of the free vaccination campaign on vaccination adherence for individuals at the 65‐year age threshold. The continuity in individual characteristics at the 65‐year‐old threshold is tested in Table 1 for both samples, the whole sample and the threshold‐based sample and is presented graphically in the Appendix (see Supporting Information S1: Figures D1 to D6 for the whole sample and Supporting Information S1: Figures D7 to D11 for the threshold‐based sample in the appendix). It is also assessed by estimating a transformed version of Equation (4), where each individual characteristic is used as a dependent variable (see Supporting Information S1: Tables D1 to D4 for the whole sample and Tables D5 to D8 for the threshold‐based sample, in the Appendix).

Approximately 84% of the individuals in the sample are in a relationship, 49% are male in the whole sample (resp. 45% in the threshold‐based sample), and 81% (resp. 84%) have a high‐school diploma. These proportions are consistent on both sides of the age threshold, with no discontinuity at the 65‐year‐old threshold (see Supporting Information S1: Figures D1 and D7 and, Tables D1 and D5 in the appendix). There is also no discontinuity in the socio‐professional category (see Supporting Information S1: Figures D2 and D8 and Tables D2 and D6 in the appendix). Not surprisingly, we observe a higher percentage of retired individuals among the treated than among the untreated (+13% points), but there is no discontinuity in the probability of retiring at the age of 65, as shown in Supporting Information S1: Figures D3 and D9 and in Tables D2 and D6 in the Appendix. Our identification strategy is valid only if the opportunity cost of vaccination uptake is not affected by retirement; we will discuss this hypothesis in further detail in Section 4.4.3. We also observe a small difference in the proportion of individuals with chronic diseases (37% among treated vs. 29% among untreated) but, as already discussed (Section 4.2), this proportion does not vary discontinuously at the threshold (see also Table D12 in the Appendix).

The average household size and the average net income are the same on each side of the threshold, and there is no discontinuity in these variables (see Supporting Information S1: Figures D5 and D10 and, Tables D3 and D7). Regarding individual preferences, Table 1 shows that 55% (resp. Fifty‐three percent) of individuals are risk‐averse in the whole (resp. the threshold‐based) sample, and this proportion remains the same around the age threshold (see also Figures D6 and D11 and, Tables D4 and D8 in the Appendix).

#### 4.4.2 Absence of Manipulation of the Running Variable

Another identifying assumption is the absence of manipulation of the running variable. Although it appears unlikely in our case that individuals would falsify their age to the French NHI to access the free vaccination program, we follow McCrary (2008) and examine the density of observations around the threshold. This density does not exhibit any discontinuity; on both samples, there is no evidence of manipulation of the running variable (see Supporting Information S1: Figure D12 in the appendix).

#### 4.4.3 Retirement and Vaccination Take‐Up

The estimation strategy requires the absence of any other policy change at the age of 65 that could explain the increase in vaccination take‐up. In France, there is no specific public policy that targets individuals over the age of 65 years, such as those concerning health expenditures. However, turning 65 coincides with significant life changes, including a high probability of retirement and therefore an increase in free time. If there were an increase in the probability of retirement at age 65, the estimated effect on vaccination take‐up could be attributed to leaving the job market.

In France, in 2014, retirement occurred mostly around the age of 60, as shown in Figure D13 in the Appendix. The proportion of retired individuals increases from 20% to 40% between ages 59 and 60, and from 40% to approximately 70% between ages 60 and 61. Between 61 and 65 years of age, this proportion continues to increase, but at a much slower rate, and there is no discontinuity in this proportion at 65 years of age (as shown in Supporting Information S1: Figure D3 and Table D2). If retirement had an effect on vaccination, we would expect a significant increase in the probability of vaccination at ages 60 and 61, when the probability of retirement increases sharply. We test this hypothesis by running placebo tests, that is, estimating reduced‐form regressions (see Equation (4)) using different age thresholds (Supporting Information S1: Table D9 in the appendix). There is no significant increase in the probability of vaccination at ages 60, 61, 62, 63, or 64. Retirement does not coincide with an increase in vaccination.

The age of 65 also marks the point at which individuals start receiving their full pension. However, as mentioned earlier, there is no significant difference in the average income level between treated and untreated individuals, and there is no discontinuity in income at the age of 65 (see Table 1, Supporting Information S1: Figure D5, and Table D3 in the Appendix).

In general, the discontinuity in vaccination rates observed at 65 is unlikely to be caused by a change in employment status (resulting in increased free time) or a change in income.

#### 4.4.4 Placebo Tests on Individuals With Chronic Diseases

In the threshold‐based sample, individuals with chronic diseases are removed. However, they constitute an interesting placebo group. Indeed, for them, we should not observe any change in the probability of being vaccinated against the flu at age 65, which is the case, as shown in Figure D14 in the Appendix. Table D10 confirms that there is no significant impact of turning 65 in this subpopulation (−0.09, se = 0.09).

In the econometric analysis, we present the estimation results separately for the two samples: the whole sample and the one without the chronically ill individuals, that is, the threshold‐based sample. We emphasize the latter; for individuals in the threshold‐based sample, there is a notable shift in vaccination incentives.

## 5 Results

### 5.1 Main Results

We first evaluate the causal impact of receiving the voucher and the leaflet on vaccination behavior. Results are presented in Table 2 for the whole sample (columns 1–3) and for the threshold‐based sample (columns 4–6).

**TABLE 2:** Regression discontinuity estimates of Equations ((2), (3), (4)), using a bandwidth of 44 months around the 65 years old threshold, on the two different samples.

	Whole sample			Threshold‐based sample		
	First stage	Second stage	Reduced form	First stage	Second stage	Reduced form
	*p*(Invit = 1)	*p*(Vacc = 1)	*p*(Vacc = 1)	*p*(Invit = 1)	*p*(Vacc = 1)	*p*(Vacc = 1)
	(1)	(2)	(3)	(4)	(5)	(6)
**Non‐parametric specification**						
$1_{A_{i}\geq 65}$	0.40***	—	0.06	0.49***	—	0.12**
se	(0.05)		(0.05)	(0.05)		(0.05)
Invitation	—	0.16	—	—	0.25**	—
se		(0.11)			(0.10)	
**Parametric specification**						
$1_{A_{i}\geq 65}$	0.44***	—	0.06	0.54***	—	0.12***
se	(0.04)		(0.04)	(0.05)		(0.05)
Invitation	—	0.14	—	—	0.22***	—
se		(0.09)			(0.08)	
*N*	1698	1698	1698	1130	1130	1130

*Note:* Standard errors in parentheses. ***Statistically significant at the 1% level; **Statistically significant at the 5% level; *Statistically significant at the 10% level. For the non‐parametric specification, conventional coefficients with conventional RD estimates are presented. For the parametric specification, we control for linear trends of age, continuous at the age of 65: $\left(A_{i}-65\right)1_{A_{i}\geq 65}\;\text{and}\;\left(A_{i}-65\right)1_{A_{i}<65}$.

*Source:* ESPS 2014.

On the whole sample, first stage estimates (column 1) show an increase of 40–44 pp in the probability of individuals reporting receipt of the invitation. In other words, the vaccination policy effectively informs individuals about their eligibility. In this sample, we observe a non‐significant increase in vaccination uptake of 14–16 pp. Similarly, in reduced form, turning 65 has a positive impact of 6 pp on the probability of vaccination, though it is not statistically significant.

Restricting the sample to individuals without chronic diseases, we find that the probability of receiving the invitation increases between 49 and 54 pp at the age threshold, depending on the specification (Column 4, Table 2). This first stage shows a very large effect on the probability of receiving the treatment, which is crucial for estimating τ (see Equation (4)): the denominator must not be 0. The impact of receiving the invitation on vaccination uptake is positive, larger than previously observed, and now significant at the 1% level in all specifications (+22 or +25 pp). Compared to the average vaccination take‐up among untreated individuals (14%), it leads to a doubling of the vaccination uptake. The reduced form estimates (Table 2, Column 6) also indicate a +12 pp increase in vaccination. These effects are obtained using a conventional variance estimator. However, bias‐corrected coefficients, along with conventional and robust corrected standard errors, are presented in the Appendix (see Supporting Information S1: Table E1) and lead to very similar results. Numerous robustness checks will be presented in Section 5.3 (use of other bandwidths, of different specifications, of restricted samples, inclusion of control variables). They all confirm these findings.

Overall, we observe a clear and significant increase in vaccination rates due to information on eligibility for free vaccination, demonstrating the effectiveness of the campaign on compliers. Nevertheless, the overall vaccination rate for those aged 65 and older remains far below the WHO's target of vaccinating 75% of the population over 65 years old.

Our model allows us to estimate the causal effect of the campaign on vaccination, that is of awareness regarding eligibility for free vaccination and on the risks associated with the flu. It is not possible to distinguish in the positive effect of the campaign the portion that comes from a price effect (vaccination is free thanks to the voucher) from the portion resulting from an informational effect (individuals have a better understanding of the disease and its associated risks thanks to the leaflet). However, as explained above, the total cost of vaccinating is relatively low (9 euros). Moreover, an analysis of the heterogeneity of the campaign's effects based on individuals' income levels reveals no significant difference in reactions between poorer individuals (those with income below the median) and richer individuals (those with income above the median). These two observations suggest that the informational effect may outweigh the price effect, but a more in‐depth analysis, which we cannot conduct due to a lack of adequate data, is necessary to confirm this hypothesis.

### 5.2 Effectiveness of the Campaign Depending on Risk Aversion

#### 5.2.1 Heterogeneity in the Effect of the Campaign

We then investigate the heterogeneity in the reaction of individuals to the campaign according to their level of risk aversion. An individual is considered risk‐averse if they rate between 0 and 4 on a scale from 0 to 10 regarding their attitude toward risk. Results of the estimates of Equation (5) are presented in Table 3 ^5^. First, risk‐averse individuals do not significantly declare more than risk takers receiving the invitation letter (first‐stage estimates are presented in Table E2 in the Appendix). Both types of individuals are aware of their eligibility. However, risk‐averse individuals react more than others to vaccination incentives. The invitation does not significantly increase the vaccination rate of risk‐takers at the 5% level (see Table 3). In contrast, risk‐averse individuals experience an increase in vaccination of 12–16 pp.

**TABLE 3:** Regression discontinuity estimates of Equation (5), using a bandwidth of 44 months around the 65 years old threshold, on the two different samples—Heterogeneity according to risk aversion.

	Whole	Threshold‐based
	sample	sample
	*p*(Vacc = 1)	*p*(Vacc = 1)
**Non‐parametric specification**		
Invitation	0.10	0.17
se	(0.12)	(0.10)
Invitation × risk‐averse	0.13	0.16**
se	(0.08)	(0.08)
Risk‐averse	−0.03	0.00
se	(0.05)	(0.04)
**Parametric specification**		
Invitation	0.08	0.13
se	(0.10)	(0.09)
Invitation × risk‐averse	0.12*	0.15**
se	(0.07)	(0.07)
Risk‐averse	−0.03	−0.01
se	(0.04)	(0.04)
*N*	1698	1130

*Note:* Standard errors in parentheses. ***Statistically significant at the 1% level; **Statistically significant at the 5% level; *Statistically significant at the 10% level. For the non‐parametric specification, conventional coefficients with conventional RD estimates are presented. For the parametric specification, we control for linear trends of age, continuous at the age of 65: $\left(A_{i}-65\right)1_{A_{i}\geq 65}\;\text{and}\;\left(A_{i}-65\right)1_{A_{i}<65}$. These linear trends are not interacted with the risk aversion dummy, as the interaction terms were not significant. Two first‐stage equations are estimated (results are presented in Table E2 in the Appendix): one with $R_{i}$ as the dependent variable and one with $R_{i}*RA$ as the dependent variable.

*Source:* ESPS 2014.

The heterogeneity according to risk aversion is particularly noteworthy given that we find no significant differences in reactions to vaccination incentives across usual individual characteristics such as gender, marital status, education, or income (see Tables E3 in the Appendix). Furthermore, using information on individual geographical location, we also find no significant differences based on the density of general practitioners in their area of residence, nor based on the severity of the last influenza epidemic (measured by hospitalizations, deaths, or emergency room visits due to influenza).

#### 5.2.2 Characteristics of the Never‐Takers

To further investigate the impact of risk aversion on the response of individuals to the vaccination campaign, we adopted the methodology proposed by Marbach and Hangartner (2020), previously used by Dynarski et al. (2021), de et al. (2021), and Merida and Rocha (2021). Under the assumption that there are no defiers, this approach allows us to distinguish between compliers and non‐compliers using an instrumental variable, specifically the age threshold of 65 years in our study. Compliers in our context are individuals aged 65 and older who were vaccinated due to the campaign but would not have been vaccinated otherwise. Always‐takers (resp. never‐takers) are individuals who are consistently vaccinated (resp. never vaccinated) regardless of the age threshold.

**FIGURE 5:**
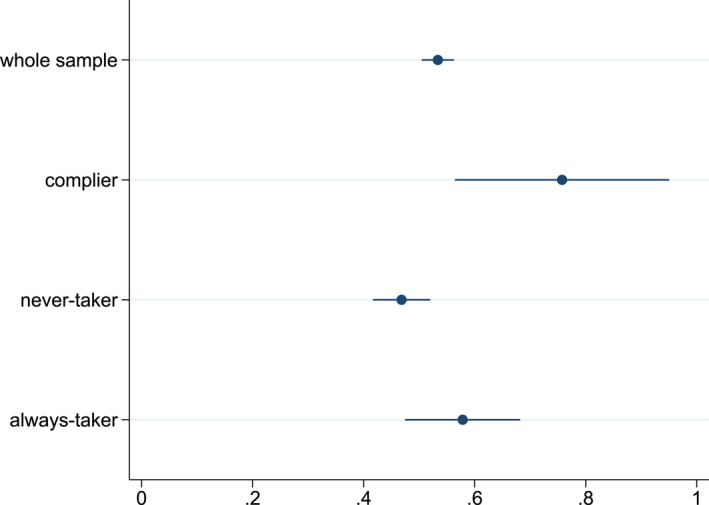
Observed proportion of risk‐averse individuals in the threshold‐based sample, and estimated proportions among the sub‐populations of compliers, never‐takers and always takers. The proportions of risk‐averse individuals in all groups are estimated using the approach presented in Marbach and Hangartner (2020). Bootstrapped 95% confidence intervals are provided. *Source:* ESPS 2014.

Figure 5 presents the percentage of risk‐averse individuals among compliers, never‐takers and always‐takers. We observe a significant difference in the percentage of risk‐averse individuals between compliers and never‐takers. More than 75.7% of the compliers are risk‐averse, while this is the case for only 46.6% of the never‐takers. Note that in the Appendix, Supporting Information S1: figures E1 show that there are no differences between compliers and never takers (or always takers) in terms of marital status, gender, education level or income, a result that is consistent with the absence of heterogeneous effects based on sociodemographic characteristics.

### 5.3 Robustness Checks

Our results are robust to a range of robustness checks.

#### 5.3.1 Use of Other Specifications

First, Supporting Information S1: Table F1 in the Appendix shows that our results are robust to the inclusion of control variables. The effect (using the threshold‐based sample) is estimated to be about 26 pp (compared to 25 pp without controls). Second, one might think that people living in the same county share similar experiences, such as previous flu epidemics. This non‐independence among individuals within the same area should be considered in the regressions. Therefore, we cluster standard errors by county and the results remain robust to this clustering. Finally, Table F1 also shows that our results remain stable when we use a non‐parametric local quadratic specification, instead of a linear one.

#### 5.3.2 Use of Other Bandwidths

**FIGURE 6:**
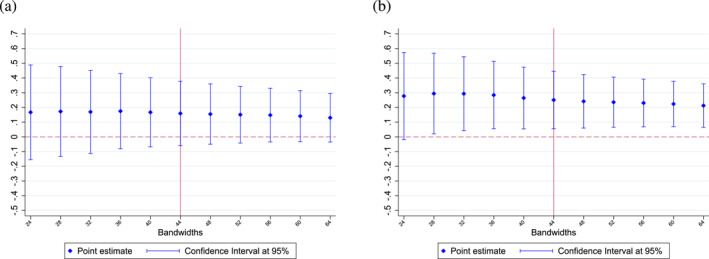
Regression discontinuity estimates of Equation (3), using different bandwidths. (a) Point estimates using different bandwidths ‐ whole sample. (b) Point estimates using different bandwidths ‐ threshold‐based sample. Estimates are obtained using a non‐parametric specification; 95% confidence intervals are presented. *Source:* ESPS 2014.

**FIGURE 7:**
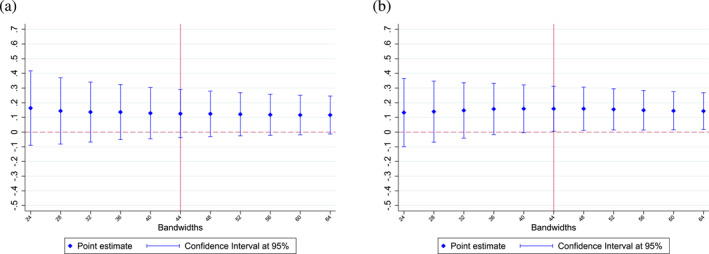
Regression discontinuity estimates of Equation (5), using different bandwidths ‐ point estimates for risk averse individuals. (a) Point estimates using different bandwidths ‐ whole sample. (b) Point estimates using different bandwidths ‐ threshold‐based sample. Estimates are obtained using a non‐parametric specification; 95% confidence intervals are presented. *Source:* ESPS 2014.

We also observe similar results when using narrower (24–42 months) or broader (46–64 months) bandwidths (see Figure 6). As shown in Table 2, our results on vaccination rates are not significant when focusing on the whole sample. However, the effect on flu vaccination uptake remains robust regardless of the bandwidth chosen on the threshold‐based sample. Furthermore, the higher response of risk‐averse individuals is also consistent across different bandwidths (see Figure 7). First‐stage and reduced‐form estimates are also robust to the choice of the bandwidth (see Supporting Information S1: Figures F1 and F2 in the appendix). Overall, our results are not dependent on the choice of the optimal bandwidth following the procedure proposed by Calonico et al. (2014).

#### 5.3.3 Placebo Tests Using Different age Thresholds

One possible placebo test involves running regressions on vaccination rates using placebo cut‐offs, such as using cut‐offs at ages 60–69 years old, as suggested by Barreca et al. (2016). As shown in Section 4.4.3 we do not find any significant change in vaccination behavior at the thresholds 60 to 64 (see Supporting Information S1: Table D9 in the appendix). Moreover, we do not observe any significant changes when using ages 66 to 69 as thresholds (refer to Supporting Information S1: Tables F2 and F3 in the Appendix). The increase in vaccination is only observed at the age cut‐off of 65 years old and not at any other cut‐offs.

#### 5.3.4 Alternative Measure of Risk Aversion

In the main analysis, individuals who declared values of 5 and above to the question on risk aversion outlined in Supporting Information S1: Table B1 in the Appendix were considered “risk takers”, while those who answered 4 and below were classified as “risk‐averse”. However, to account for the focal point at the value of “5” (as observed in Figure B2 in the Appendix), we checked the sensitivity of our results by including the focal answer among “risk‐averse” individuals and defining “risk‐averse” as individuals who report values of 5 and below. Our findings remain consistent when using this alternative measure of risk aversion, with an increase of 15–18 pp in vaccination rates, only for risk‐averse individuals (see Supporting Information S1: Table F4 in the Appendix).

#### 5.3.5 Robustness Checks on Restricted Samples

Individuals born between October and December 1949 have reached the age of 65 during the vaccination campaign. Although it is improbable that these individuals were left untreated,^6^ there is a chance that some of them did not receive the invitation letter by the time of the interview. To ensure accurate identification of the treatment, we employ a restricted sample that excludes these individuals (see Supporting Information S1: Tables F5 and F6 in the Appendix). The results align closely with those observed in the main analysis, leading us to conclude that the definition of the treatment variable is accurate.

Another potential source of confusion lies in the interpretation of the vaccination behavior question. Participants are asked to report their vaccination status during the *last* vaccination campaign. Consequently, individuals surveyed from October 2014 onward might have indicated their vaccination behavior for the ongoing campaign (2014/2015) rather than the campaign we are studying (2013/2014) and for which we have defined our treatment. This discrepancy could lead to an underestimation of the effects. To mitigate this issue, we have excluded all individuals interviewed from October 2014 onward, resulting in the removal of approximately 700 individuals. The results mirror those presented in Section 5 (see Supporting Information S1: Tables F7 and F8 in the Appendix).

Finally, the receipt of the invitation letter by an eligible spouse could trigger a spillover effect on the non‐eligible spouse. The non‐eligible spouse might alter their vaccination behavior without directly receiving the invitation, which could result in an underestimation of the invitation's effects. We do not have enough observations in our sample to test whether their exists a spillover effect on younger spouses, as done in Bouckaert et al. (2020). However, to address this issue, we analyze a sample comprising individuals from households where all members fall on the same side of the threshold. This approach ensures the elimination of any spillover effects. The results displayed in Supporting Information S1: Tables F9 and F10 closely resemble those in Section 5, suggesting that our conclusions are not significantly underestimated as a result of potential spillover effects.

## 6 Theoretical Mechanisms

To understand why risk‐averse individuals may be more sensitive to the vaccination campaign, we present a simple model. We model a vaccination decision made by an expected utility‐maximizing decision maker who is exposed to two possible actions: getting vaccinated against the flu or not. If the individual decides to get vaccinated (option V), she will face a probability $p_{s}$ of complications due to the injection of the vaccine. She will pay a cost $c_{s}$, which includes the monetary and non‐monetary cost of the vaccine (price of the vaccination, time or monetary opportunity cost). If we normalize the utility of being healthy to u(0), then the expected utility of being vaccinated is:

(6) $$Eu\left(V\right)=p_{s}u\left(-c_{s}\right)+\left(1-p_{s}\right)u\left(0\right)$$

If the individual decides not to be vaccinated (option NV), she will face a probability $p_{i}$ of getting infected by the flu. The associated cost (disease, possible complications, etc.) is denoted $c_{i}$. The expected utility of not being vaccinated is:

(7) $$Eu\left(NV\right)=p_{i}u\left(-c_{i}\right)+\left(1-p_{i}\right)u\left(0\right)$$

Let u(x) be a CARA (Constant Absolute Risk Aversion) utility function where β>0 measures the risk aversion: a higher β corresponds to a higher absolute level of risk aversion. We denote $u\left(x\right)=-e^{-\beta x}$.

An individual opts for vaccination if and only if:

(8) $$\begin{array}{cc} Eu\left(V\right)\geq Eu\left(NV\right) & ⟺p_{s}u\left(-c_{s}\right)+\left(1-p_{s}\right)u\left(0\right)\geq p_{i}u\left(-c_{i}\right)+\left(1-p_{i}\right)u\left(0\right)\\ & ⟺-p_{s}e^{\beta c_{s}}-1+p_{s}\geq -p_{i}e^{\beta c_{i}}-1+p_{i}\\ & ⟺\underset{f\;\left(\beta \right)}{\underbrace{-p_{s}e^{\beta c_{s}}+p_{i}e^{\beta c_{i}}}}\geq p_{i}-p_{s} \end{array}$$

Denoting $f\left(\beta \right)=-p_{s}e^{\beta c_{s}}+p_{i}e^{\beta c_{i}}$, an individual gets vaccinated if $f\left(\beta \right)\geq p_{i}-p_{s}$ and does not get vaccinated if $f\left(\beta \right)<p_{i}-p_{s}$. To analyze the values of β that lead to vaccination or non vaccination, we study the function f(β). With $\overset{∼}{\beta }$ being the solution of $f\left(\overset{∼}{\beta }\right)=p_{i}-p_{s}$, the optimal choice of vaccination can be summarized in the Table below.

Proposition 1 Before receiving the invitation, the individuals' optimal choice of vaccination is.

**Table:** 

	$c_{s}-c_{i}>0$	$c_{s}-c_{i}<0$
$\frac{p_{i}c_{i}}{p_{s}c_{s}}<1$	(1) NV ∀β	(2) V $\Leftrightarrow \;\beta >\overset{∼}{\beta }$
$\frac{p_{i}c_{i}}{p_{s}c_{s}}>1$	(3) V $\Leftrightarrow \;\beta <\overset{∼}{\beta }$	(4) V ∀β

*Proof*. We observe that $\lim_{\beta \to 0}f\left(\beta \right)=p_{i}-p_{s}$; $\lim_{\beta \to \infty }f\left(\beta \right)=-\infty$ if $c_{s}>c_{i}$; $\lim_{\beta \to \infty }f\left(\beta \right)=\infty$ if $c_{s}<c_{i}$.

Moreover, $f^{\prime }\left(\beta \right)=-p_{s}c_{s}e^{\beta c_{s}}+p_{i}c_{i}e^{\beta c_{i}}$ so that $f^{\prime }\left(\beta \right)>0\;⟺\;\ln\frac{p_{i}c_{i}}{p_{s}c_{s}}>\beta \left(c_{s}-c_{i}\right)$. The solution depends on whether $c_{s}-c_{i}$ is positive or negative and whether $\ln\frac{p_{i}c_{i}}{p_{s}c_{s}}$ is lower or higher than 1 (detailed calculations are presented in Supporting Information S1: Appendix G).

Cases 1 and 4 are unrealistic: they conclude that no one is getting vaccinated before the invitation (Case 1) or everyone is getting vaccinated (Case 4), whatever their level of risk aversion, while neither of these situations is observed in practice (Vaux et al. 2011). Conversely, Cases 2 and 3 are realistic. Case 2 (resp. Case 3) means that individuals with higher (resp. lower) levels of risk aversion get vaccinated. We therefore concentrate on those two cases to analyze how the receipt of the invitation letter changes individuals' behavior.

By informing individuals about the risks of the disease, the leaflet changes individuals' initial values of $c_{i}$ and $p_{i}$ and the voucher changes the value of $c_{s}$. However, the invitation letter has no impact on $p_{s}$. Lets denote $\left(c_{i},p_{i}\right)$ as the cost of contracting the disease and the probability of contracting it before receiving the invitation letter and $\left(c_{i}^{\prime },p_{i}^{\prime }\right)$ those after the receipt of the invitation, such that $c_{i}^{\prime }\geq c_{i}$ and $p_{i}^{\prime }\geq p_{i}$. Similarly, let us denote $\left(c_{s},p_{s}\right)$ as the cost of the vaccine and the probability of complications due to the injection and $\left(c_{s}^{\prime },p_{s}\right)$ those after the receipt of the letter, such that $c_{s}^{\prime }<c_{s}$. Let g be a function such that $g\left(\beta ,c_{i}^{\prime },p_{i}^{\prime },c_{s}^{\prime }\right)=-p_{s}e^{\beta c_{s}^{\prime }}+p_{i}^{\prime }e^{\beta c_{i}^{\prime }}+p_{s}-p_{i}^{\prime }$. The individual decides to be vaccinated if and only if $g\left(\beta ,c_{i}^{\prime },p_{i}^{\prime },c_{s}^{\prime }\right)\geq 0$. We show in Appendix I that g is increasing with $c_{i}^{\prime }$, $p_{i}^{\prime }$ and $c_{s}^{\prime }$.

In Case 2, that is when $\frac{p_{i}c_{i}}{p_{s}c_{s}}<1$ and $c_{s}-c_{i}<0$, $c_{s}^{\prime }-c_{i}^{\prime }$ remains negative when the invitation is received as $c_{i}^{\prime }\geq c_{i}$ and $c_{s}^{\prime }<c_{s}$.

Thus, if $\frac{p_{i}^{\prime }c_{i}^{\prime }}{p_{s}c_{s}^{\prime }}$ becomes greater than 1, then the agent vaccinates whatever β.

If $\frac{p_{i}^{\prime }c_{i}^{\prime }}{p_{s}c_{s}^{\prime }}$ remains below 1, as g increases with $\left(c_{i}^{\prime },p_{i}^{\prime },c_{s}^{\prime }\right)$, we get: $g\left(\overset{∼}{\beta^{\prime }},c_{i}^{\prime },p_{i}^{\prime },c_{s}^{\prime }\right)\geq g\left(\overset{∼}{\beta^{\prime }},c_{i},p_{i},c_{s}\right)=0$.^7^ By definition of $\overset{∼}{\beta^{\prime }}$, $g\left(\beta^{\prime },c_{i}^{\prime },p_{i}^{\prime },c_{s}^{\prime }\right)<0$ for all $\beta^{\prime }<\overset{∼}{\beta^{\prime }}$. Therefore, $\overset{∼}{\beta^{\prime }}\leq \overset{∼}{\beta }$. As a result, agents such as β > $\overset{∼}{\beta }$ get vaccinated whether they receive the invitation or not. And agents with $\beta \in \left[\overset{∼}{\beta^{\prime }};\overset{∼}{\beta }\right]$ do not get vaccinated if they don't receive the invitation letter but get vaccinated if they do. In other words, among those who don't vaccinate without the invitation letter, it is the most risk‐averse who vaccinate if they receive the invitation.

A similar reasoning can be made for Case 3 (see Appendix G for more details). In that case, we find that among individuals who did not vaccinate before the invitation letter, it is the risk‐takers who vaccinate if they receive the letter.

Intuitively, both cases are possible: (i) on receipt of the invitation, risk‐averse individuals are vaccinated being more receptive to the vaccination campaign and to the information on the risks of the disease described in the letter; (ii) on receipt of the invitation, risk‐taker individuals are more likely to be vaccinated as they have not been vaccinated before, which would be a potential catch‐up effect.

Results of the econometric analysis show that only the most risk‐averse individuals respond to the vaccination incentives created by the campaign, which is in line with Case 2 of our theoretical model. More precisely, the vaccination campaign lowered down the level of risk aversion from which risk‐averse individuals get vaccinated. For these individuals, the model shows that the fear of contracting the flu outweighs concerns about potential side‐effects. Our empirical results contradict Case 3, meaning that there is no catch‐up effect following invitation reception for risk‐taker individuals. For them, the costs of the side effects of the vaccine (such as wasted time, pain, perceived uselessness, etc.) outweigh the costs associated with contracting the flu. The reduction in monetary costs after the campaign is insufficient to compensate for all non‐monetary costs associated with receiving the vaccine injection.

## 7 Discussion and Conclusion

Once a year, in Autumn, a national flu vaccination program is organized in France: all individuals aged 65 and more receive a voucher for free vaccination, together with a leaflet informing them about the necessity to get vaccinated and the danger of influenza. We measure the causal effect of this vaccination program on the vaccination decision for individuals aged 65 and above.

The estimates indicate a strong impact of the campaign on individuals' awareness of their eligibility for free vaccination. The probability of reporting receiving the invitation letter is 49–54 pp higher for individuals aged 65 and above. The receipt of the invitation then leads to a clear and significant increase in the probability of getting vaccinated against the flu: + 22 to 25 pp, demonstrating the effectiveness of the campaign on compliers. However, the overall vaccination rate for those aged 65 and older remains below the WHO's target of vaccinating 75% of the population over 65 years of age against the flu. In France, 2000 deaths among those over 65 years of age were estimated to have been prevented in 2014 due to vaccination. However, 3000 could have been avoided if vaccination coverage had reached the 75% target set by the WHO (Ministry of Health 2014). Therefore, it is possible to improve the effectiveness of the vaccination campaign. These results pertain to the 2013–2014 flu vaccination campaign. However, in terms of external validity, it is important to note that the year 2014 is not a standalone year. The previous years, 2012 and 2013, did not exhibit notably low flu prevalence rates, which could have influenced individuals' perception of the risk of the disease. Furthermore, there were no significant events in 2014 (such as the H1N1 epidemic) that could have deterred individuals from getting vaccinated that year.

One limitation of the study is that it is not possible to distinguish, in the positive effect of the campaign, the portion that comes from a price effect (vaccination is free thanks to the voucher) from the portion resulting from an informational effect (individuals have a better understanding of the disease and its associated risks thanks to the leaflet). Both the low total cost of getting vaccinated (around 9 euros) and the absence of any heterogeneity in individuals' responses based on income suggest that the informational effect likely outweighs the price effect. However, this hypothesis should be tested more thoroughly in future research.

Another significant finding is that the positive impact of the campaign is primarily driven by more risk‐averse individuals; risk‐takers do not demonstrate a response to the invitation letter. Vaccination decisions are made by weighing the risk of vaccine‐related side effects against the risk of contracting the flu. Our theoretical framework suggests that the higher response of risk‐averse individuals aligns with a scenario where the costs associated with vaccine side effects are lower than the costs of flu contraction. In this context, the most risk‐averse individuals alter their behavior after receiving the invitation. For risk takers, influenza may not pose a significant threat, and the reduction in monetary expenses through the voucher may not offset the non‐monetary time costs. Through the analysis, we contribute to a deeper understanding of the underlying motivations of individuals that influence their vaccination decisions, such as risk aversion. One limitation of this finding is that the survey question on risk aversion relates to eliciting individual attitudes toward risk aversion in life in general, while we hypothesized that it is risk aversion toward health behavior. Therefore, these findings should be confirmed using a specific measure of risk aversion related to health.

In the realm of public health policies, efforts to inform individuals about their eligibility for free vaccination and educate them about the health risks of influenza can effectively enhance vaccination rates, although this alone may not be sufficient. The heterogeneity in behaviors according to risk aversion underscores the importance of targeted approaches toward specific groups, particularly risk‐taker individuals, rather than employing broad strategies for the entire vulnerable population. Given that these individuals are not identifiable through the NHI system, general practitioners (GPs) could potentially play a crucial role because they are more likely to be familiar with their patients' health behaviors. The results of a 2010 survey on French physicians reveal that 90% believe they are effective in improving patient adherence to flu vaccination (although only 38% endorse making this vaccination mandatory). Furthermore, 79% of physicians regularly engage in discussions with their patients about the benefits and risks of vaccination. Financial incentives have been provided to general practitioners since 2012 to encourage vaccination among 65‐year‐old patients. However, the impact of this pay‐for‐performance initiative has been relatively limited so far. In fact, in 2016, only 0.9% of GPs had reached the target of vaccinating 75% of their patients 65 years and older (Cour des comptes, 2018). Hence, more thought is needed to design effective public policies aimed at encouraging the most risk‐takers to increase their vaccination rates against this potential severe disease.

## Conflicts of Interest

The authors declare no conflicts of interest.

## Supporting information


Supporting Information S1

## Data Availability

The data that support the findings of this study are available from Quetelet‐Progedo Diffusion. Restrictions apply to the availability of these data, which were used under license for this study. Data are available from http://www.progedo‐adisp.fr with the permission of Quetelet‐Progedo Diffusion.

## Endnotes

^1^
The leaflet highlighted five compelling reasons for getting vaccinated: (1) In at‐risk individuals, influenza can lead to serious or even fatal complications. (2) If I am over 65 years old or have a chronic disease, I am more vulnerable to the flu, even if I feel healthy. (3) The composition of the vaccines is adjusted each year according to circulating viruses, so I need to be vaccinated annually. (4) vaccination is the most effective way to protect myself and I should discuss this with my doctor. By getting vaccinated, I am also protecting my loved ones. (5) The vaccine is free for all at‐risk individuals, particularly those over 65 years old or those with chronic diseases.

^2^
In the final version of the model, functions f and g in Equation (5) are not interacted with risk aversion as the interacted terms were not significant.

^3^
Pregnant women are also eligible for free vaccination but they are not included in our sample as we focus on individuals aged around 65.

^4^
We present 2 figures. Figure 3 provides the average rate of invitation to flu vaccination per *age defined in years* to improve readability and compare the average proportions before and after the threshold. In Figure 3, *local sample means* are presented, for consistency with our econometric strategy. However, because each selected bin does not correspond to a specific age (in months), differences in rates observed on the graph are not so easy to comment on. The same presentation is used for Figure 4.

^5^
Continuity of individual observable characteristics is also verified in the samples of risk‐averse and risk‐taker individuals.

^6^
Note that all individuals who turn 65 during the calendar year of 2013 are supposed to receive the invitation.

^7^
Recall that $\overset{∼}{\beta }$ is the solution of $f\left(\beta \right)=p_{i}-p_{s}$.
