# Supplementary material for: Vaccination and Risk Aversion: Evidence From a Flu Vaccination Campaign
Source: Health Econ. 2025 Sep 24;35(1):36–51. doi: 10.1002/hec.70037 (PMC12680913; doi:10.1002/hec.70037)
Supplement: Supplementary file 1 — Supporting Information S1 [file HEC-35-36-s001.pdf]

Online Appendix

for

Vaccination and Risk Aversion: Evidence from a Flu Vaccination Campaign

Contents

A The French National Influenza Campaign 2013-2014: content of the letter sent by the NHI **2**

B Data **4**
B.1 Incidence of influenza per year . . . 4
B.2 Definition of the main variables of interest . . . 5
B.3 Dates of Interviews in the "Health and Social Protection Survey" 2014 . . . 5
B.4 Distribution of risk-aversion . . . 6
B.5 Definition of treated and untreated individuals . . . 6
B.6 Prevalence of chronic diseases in France . . . 6

C Vaccination-related outcomes for the threshold-based sample **8**

D Identifying assumptions **9**
D.1 Continuity in the characteristics at the age of 65 . . . 9
D.1.1 Graphical evidence on the whole sample . . . 9
D.1.2 Graphical evidence on the threshold-based sample . . . 12
D.1.3 Results of the estimates (non-parametric local linear estimates) on the whole sample . . . 14
D.1.4 Results of the estimates (non parametric local linear estimates) on the threshold-based sample . . . 16
D.2 McCrary test for continuity in the number of individuals at the age of 65 . 17
D.3 Retirement and vaccination take-up . . . 18
D.4 Placebo tests on individuals with chronic diseases . . . 19
D.4.1 Graphical evidence . . . 19
D.4.2 Results of the estimates . . . 19

E Main Results **20**
E.1 Non-parametric specification, using different methods for the construction of confidence intervals 20
E.2 First-stage estimates according to the level of risk aversion 21
E.3 Heterogeneous effects by socio-demographic characteristics 22
E.4 Profile of compliers, always-takers and never-takers according to several socio-demographic characteristics 23

F Robustness checks **24**
F.1 Use of other specifications 24
F.2 Use of other bandwidths 25
F.3 Placebo tests using different age thresholds 27
F.4 Alternative measure of risk aversion 29
F.5 Restricted samples 30
F.5.1 Sample excluding all individuals born between October and December 1949 30
F.5.2 Sample excluding all individuals surveyed during the vaccination campaign (from October 2014 onwards) 32
F.5.3 Sample restricted to individuals from households where all members fall on the same side of the threshold 34

G Theoretical framework **35**

A The French National Influenza Campaign 2013-2014: content of the letter sent by the NHI

All individuals aged 65 and over in 2013 as well as those who turned 65 during that calendar year (or who will turn 65), along with individuals with chronic diseases – i.e., at-risk individuals identifiable through the NHI system – receive a personalized letter at home from the NHI between September 17 and October 10, 2013.

The letter contains a vaccination voucher that allows for free vaccination between October 11 and January 31, 2014 (see Figure A1). Individuals must present the voucher to the pharmacist to receive the vaccine for free and they can, then, be vaccinated during a general practitioner's consultation or by a nurse for free.

The letter also contains a leaflet reminding people of the seriousness of influenza and the importance of vaccination (see Figure A2). The leaflet aims to raise awareness among at-risk individuals about the potential seriousness of influenza and the importance of vaccination as a safe and effective means of preventing the disease and its complications with the communication message "Flu is no joke. So I get vaccinated" (*"La grippe, ce n'est pas rien. Alors, je fais le vaccin"*, see Figures A2a and A2b). The leaflet highlights the importance of getting vaccinated for five compelling reasons (see Figure A2c): 1) In at-risk individuals, influenza can lead to serious or even fatal complications. 2) If I am over 65 years old or have a chronic disease, I am more vulnerable to the flu, even if I feel healthy. 3) The composition of the vaccines is adjusted each year according to circulating viruses, so I need to be vaccinated annually. 4) vaccination is the most effective way to protect myself and I should discuss this with my doctor. By getting vaccinated, I am also protecting my loved ones. 5) The vaccine is free for all at-risk individuals, particularly those over 65 years old or those with chronic diseases.

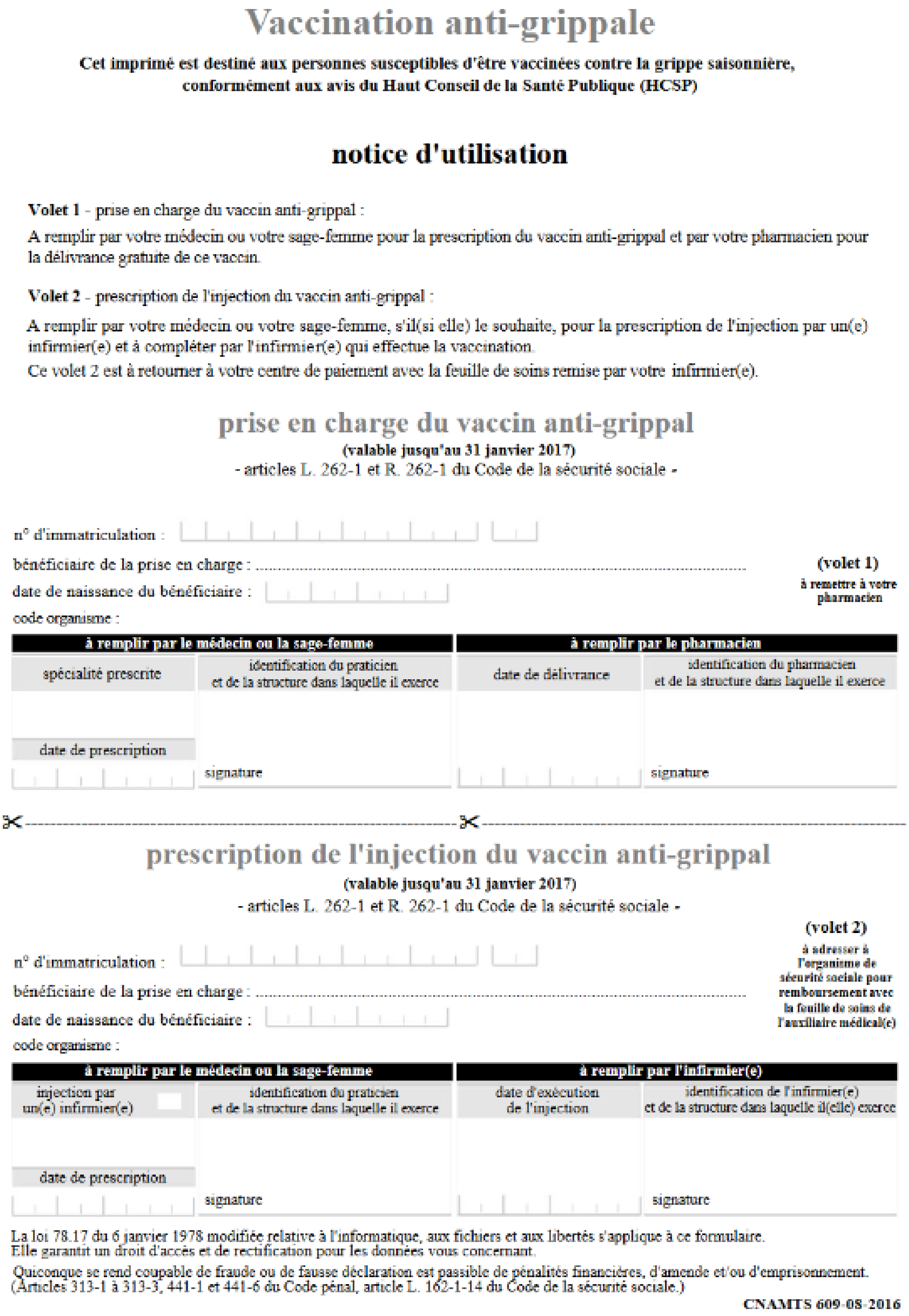

Vaccination anti-grippale

Cet imprimé est destiné aux personnes susceptibles d'être vaccinées contre la grippe saisonnière, conformément aux avis du Haut Conseil de la Santé Publique (HCSP)

notice d'utilisation

Volet 1 - prise en charge du vaccin anti-grippal :

A remplir par votre médecin ou votre sage-femme pour la prescription du vaccin anti-grippal et par votre pharmacien pour la délivrance gratuite de ce vaccin.

Volet 2 - prescription de l'injection du vaccin anti-grippal :

A remplir par votre médecin ou votre sage-femme, s'il(si elle) le souhaite, pour la prescription de l'injection par un(e) infirmier(e) et à compléter par l'infirmier(e) qui effectue la vaccination.
Ce volet 2 est à retourner à votre centre de paiement avec la feuille de soins remise par votre infirmier(e).

prise en charge du vaccin anti-grippal

(valable jusqu'au 31 janvier 2017)
- articles L. 262-1 et R. 262-1 du Code de la sécurité sociale -

n° d'immatriculation :
bénéficiaire de la prise en charge : ....
date de naissance du bénéficiaire :
code organisme :

(volet 1)
à remettre à votre pharmacien

à remplir par le médecin ou la sage-femme		à remplir par le pharmacien	
spécialité prescrite	identification du praticien et de la structure dans laquelle il exerce	date de délivrance	identification du pharmacien et de la structure dans laquelle il exerce
date de prescription	signature		signature

prescription de l'injection du vaccin anti-grippal

(valable jusqu'au 31 janvier 2017)
- articles L. 262-1 et R. 262-1 du Code de la sécurité sociale -

(volet 2)
à adresser à l'organisme de sécurité sociale pour remboursement avec la feuille de soins de l'auxiliaire médical(e)

n° d'immatriculation :
bénéficiaire de la prise en charge : ....
date de naissance du bénéficiaire :
code organisme :

à remplir par le médecin ou la sage-femme		à remplir par l'infirmier(e)	
injection par un(e) infirmier(e)	identification du praticien et de la structure dans laquelle il exerce	date d'exécution de l'injection	identification de l'infirmier(e) et de la structure dans laquelle il(elle) exerce
date de prescription	signature		signature

La loi 78.17 du 6 janvier 1978 modifiée relative à l'informatique, aux fichiers et aux libertés s'applique à ce formulaire.
Elle garantit un droit d'accès et de rectification pour les données vous concernant.
Quiconque se rend coupable de fraude ou de fausse déclaration est passible de pénalités financières, d'amende et/ou d'emprisonnement. (Articles 313-1 à 313-3, 441-1 et 441-6 du Code pénal, article L. 162-1-14 du Code de la sécurité sociale.)

CNAMTS 609-08-2016

Figure A1. Voucher accompanying the letter sent by the National Health Insurance to at-risk individuals in October-November 2017.

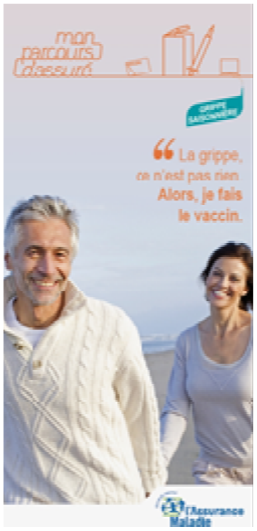

(a) Front

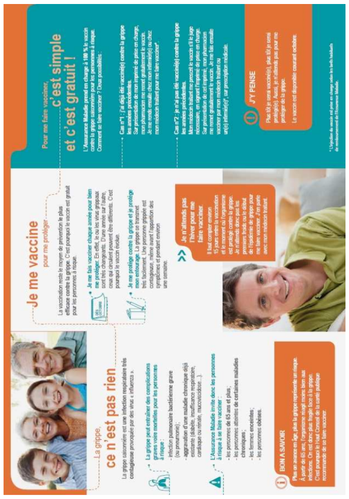

(b) Inside Left, Inside Center, Inside Right

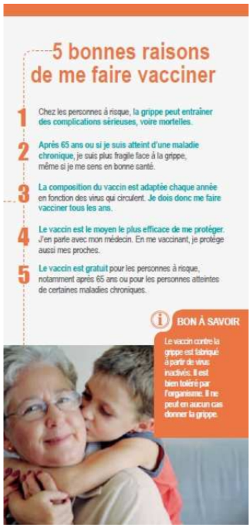

(c) Back

Figure A2. Content of the leaflet accompanying the letter sent by the National Health Insurance to at-risk individuals in October-November 2013

B Data

B.1 Incidence of influenza per year

Figure B1. Incidence of influenza per year

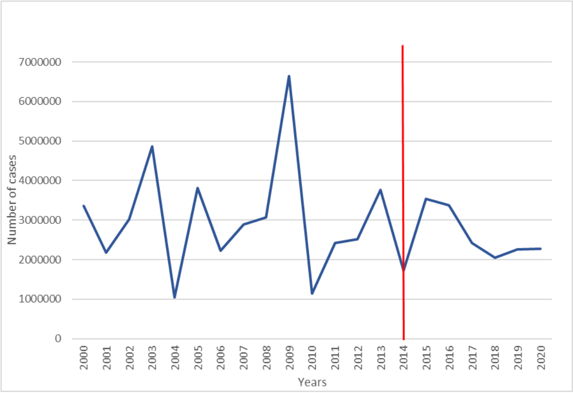

Note: Calculated by the authors using ”Réseau Sentinelles” data.

B.2 Definition of the main variables of interest

Table B2. Definition of vaccination outcomes and risk aversion

**Variable**	**Question**	**Answer**	**Values**
**Vaccination:**			
Reception of the letter	Did you receive a voucher from the NHI in the autumn of 2013 for free vaccination against the flu ?	Yes/No	1/0
Flu vaccination shot	Did you get vaccinated against the flu during the latest seasonal flu ?	Yes/No	1/0
**Preferences:**			
Risk averse	When it comes to your attitude towards risk, where do you rank on a scale from 0 to 10? (0=not at all willing to take risks / 10 = very willing to take risks)	Less than 5 vs. 5 and more	1/0

B.3 Dates of Interviews in the "Health and Social Protection Survey" 2014

Table B3. Distribution of respondents by month and year of interview

	**65 y.o. and older**			**Less than 65 y.o.**		
	2014	2015	Pct/month	2014	2015	Pct/month
January	3.52%	2.48%	6.00%	3.17%	3.68%	6.85%
February	31.04%	0.31%	31.35%	28.56%	0.59%	29.16%
March	16.65%	0%	16.65%	17.58%	0.01%	17.59%
April	3.90%	0%	3.90%	6.23%	0%	6.23%
May	2.42%	0%	2.42%	2.74%	0%	2.74%
June	0.66%	0%	0.66%	0.70%	0%	0.70%
July	0.03%	0%	0.03%	0.20%	0%	0.20%
August	0.13%	0%	0.13%	0.09%	0%	0.09%
September	11.25%	0%	11.25%	9.93%	0%	9.93%
October	12.50%	0%	12.50%	11.88%	0%	11.88%
November	8.61%	0%	8.61%	8.90%	0%	8.90%
December	6.47%	0%	6.47%	5.72%	0%	5.72%
Pct/year	97.20%	2.80%	–	95.71%	4.29%	–

Source : ESPS 2014

B.4 Distribution of risk-aversion

Figure B4. Distribution of risk aversion

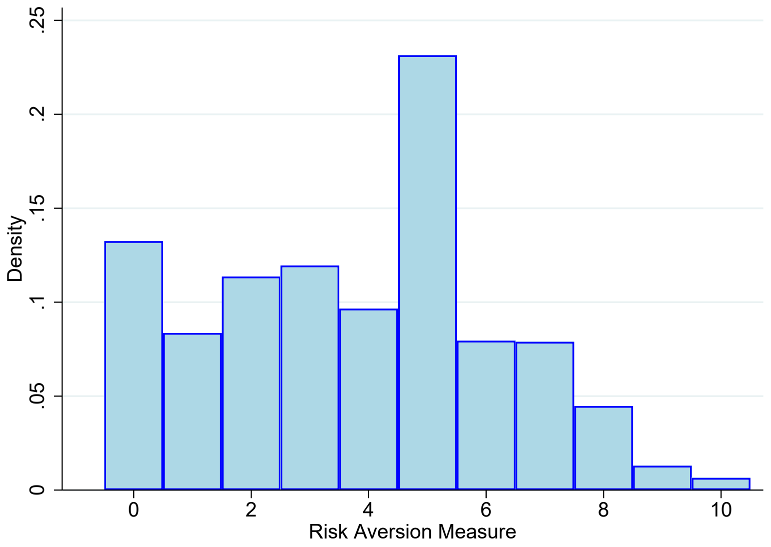

Source: ESPS 2014.

B.5 Definition of treated and untreated individuals

All individuals aged 65 and over in 2013 as well as those who turned 65 during that calendar year (or who will turn 65), are affected by the campaign: they received the voucher and the leaflet. To identify them in our sample, we use information on the month and year of birth and calculate their age in months as of December 2013.
All individuals born in December 1948 and before are between 65 years old and 68 years and 8 months old in December 2013; they are defined as treated. All individuals born in January 1949 or later are between 61 years and 4 months old and 64 years and 11 months old in December 2013: they are defined as untreated. Table B5 provides a more detailed description of treated and untreated individuals based on their date of birth.

B.6 Prevalence of chronic diseases in France

The proportion of individuals with chronic diseases in the ESPS database is very close to the percentage found at the national level in 2014. We used administrative data from the National Health Insurance (CNAMTS-DSES) on the number of individuals suffering from chronic diseases per age group (see Table B6), and data from the French National Institute for Statistical and Economic Studies (INSEE) on the population per age (see Table B7). We computed the percentage of people between the ages of 60 and 69 who suffer from chronic diseases. Approximately 27% of individuals aged 60 to 64 years and 35% of those aged 65 to 69 years suffer from chronic disease. The corresponding figures in our sample are very similar: on average, 29% of individuals aged between 61 and 64 have a chronic disease, while this is the case for 37% of individuals aged between 65 and 68.

Table B5. Definition of Treated and Untreated individuals

Distance to the cutoff (in months)	Month of Birth	Year of Birth	Age in December 2013	Treatment
-44	August	1952	61 y.o. 4 m	Non-Treated
...	...	...	...	
-6	June	1949	64 y.o. 6 m	
-5	May	1949	64 y.o. 7 m	
-4	April	1949	64 y.o. 8 m	
-3	March	1949	64 y.o. 9 m	
-2	February	1949	64 y.o. 10 m	
-1	January	1949	64 y.o. 11 m	
0	December	1948	65 y.o.	Treated
1	November	1948	65 y.o. 1 m	
2	October	1948	65 y.o. 2 m	
3	September	1948	65 y.o. 3 m	
4	August	1948	65 y.o. 4 m	
5	July	1948	65 y.o. 5 m	
6	June	1948	65 y.o. 6 m	
...	...	...	...	
44	April	1945	68 y.o. 8 m	

Table B6. Number of individuals with chronic diseases, by sex and age group in 2014 (numbers rounded to the nearest ten).

**Age**	Men	Women	Total
**60-64 y.o.**	568,750	508,570	1,077,320
**65-69 y.o.**	637,760	541,020	1,178,770

Source: National Health Insurance files - `https://www.assurance-maladie.ameli.fr/etudes-et-donnees/prevalence-beneficiaires-ald`

‘

Table B7. French population by age

Age	Men	Women	Total
**60 y.o.**	379,267	409,196	788,463
**61 y.o.**	384,482	416,965	801,447
**62 y.o.**	374,406	405,025	779,431
**63 y.o.**	389,143	420,940	810,083
**64 y.o.**	380,863	414,362	795,225
**60-64 y.o.**	**1,908,161**	**2,066,488**	**3,974,649**
**65 y.o.**	381,072	412,592	793,664
**66 y.o.**	370,112	403,527	773,639
**67 y.o.**	349,277	384,003	733,28
**68 y.o.**	259,874	290,064	549,938
**69 y.o.**	251,712	284,856	536,568
**65-69 y.o.**	**1,612,047**	**1,775,042**	**3,387,089**

Source: French National Institute for Statistics and Economic Studies - Age pyramid as of January 1, 2014 `https://www.insee.fr/fr/statistiques/1288324?sommaire=1288404#tableau-TF14032G1`.

C Vaccination-related outcomes for the threshold-based sample

Figure C1. Vaccination outcomes, by age in months on the threshold-based sample - zero cutoff for the 65 years old and bandwidth of 44 months

(a) Flu vaccination invitation rate

(b) Flu vaccination rate

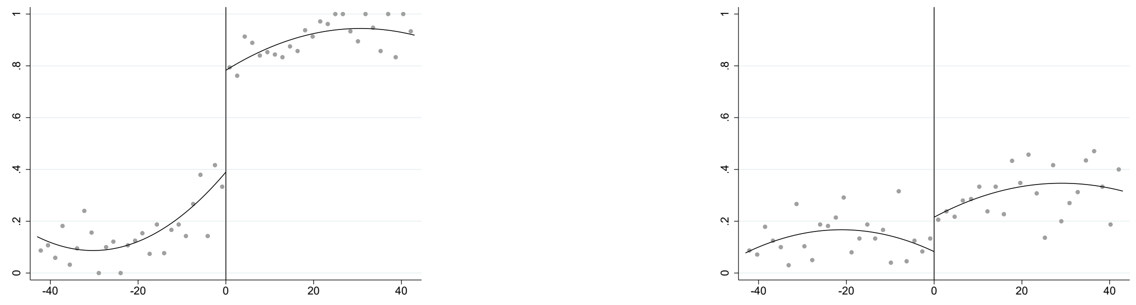

Source: ESPS 2014.

D Identifying assumptions

D.1 Continuity in the characteristics at the age of 65

D.1.1 Graphical evidence on the whole sample

Figure D1. Continuity in the socio-demographic characteristics - zero cutoff for the 65 years old and bandwidth of 44 months

(a) Percentage of individuals in a relationship by age in months

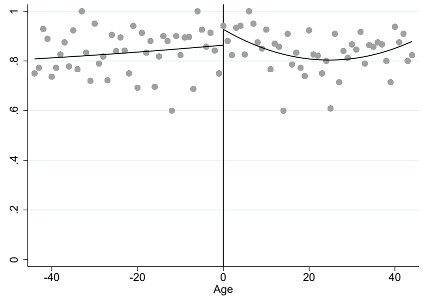

(b) Percentage of males by age in months

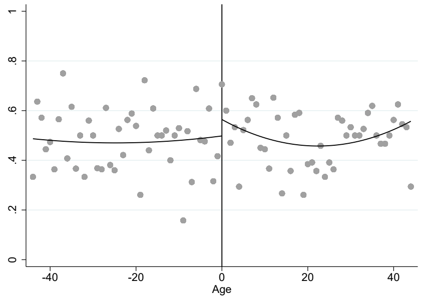

(c) Percentage of individuals with a high school diploma by age in months

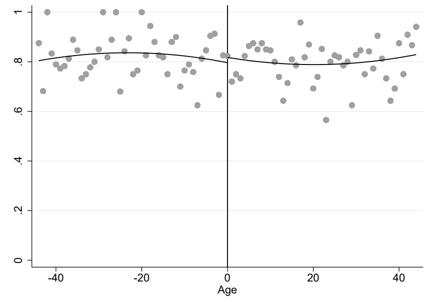

Source: ESPS 2014.

Figure D2. Continuity in the socio-professional category - zero cutoff for the 65 years old and bandwidth of 44 months

(a) Percentage of Executives by age in months

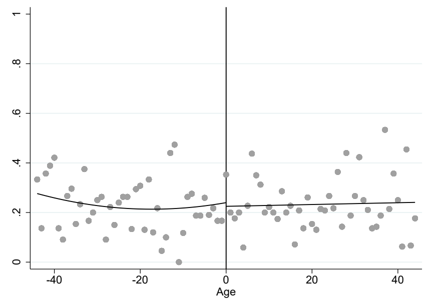

(b) Percentage of Employees by age in months

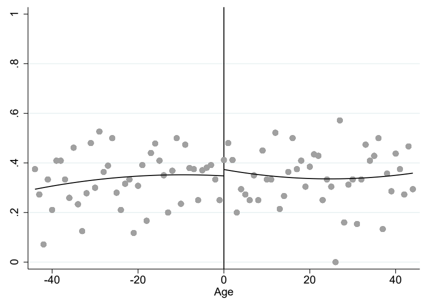

(c) Percentage of Blue Collars by age in months

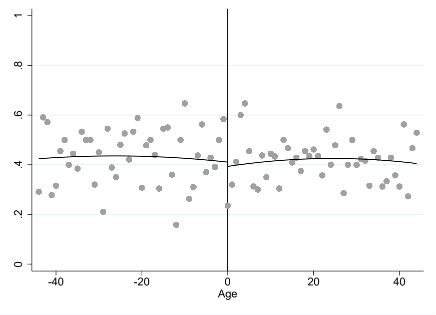

Source: ESPS 2014.

Figure D3. Continuity in the percentage of pensioners - zero cutoff for the 65 years old and bandwidth of 44 months

(a) Percentage of pensioners by age in months

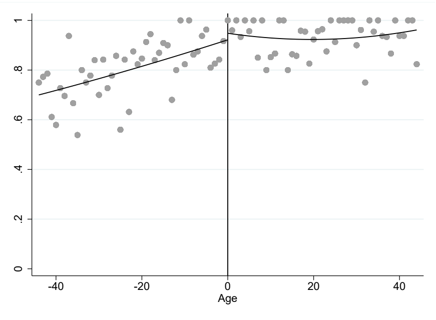

Source: ESPS 2014.

Figure D4. Continuity in the proportion of individuals chronically ill - zero cutoff for the 65 years old and bandwidth of 44 months

(a) Percentage of individuals with a chronic disease by age in months

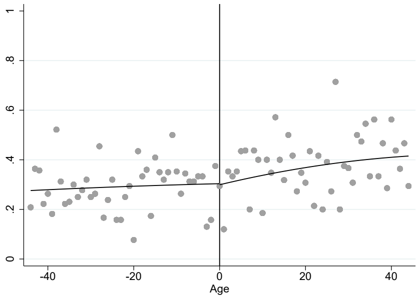

(b) Percentage of individuals eligible for free vaccination regardless of their age, by age in months

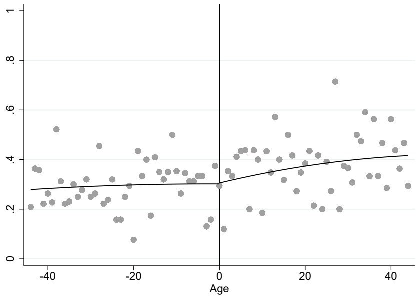

Source: ESPS 2014.

Figure D5. Continuity in household characteristics - zero cutoff for the 65 years old and bandwidth of 44 months

(a) Average number of individuals in the household by age in months

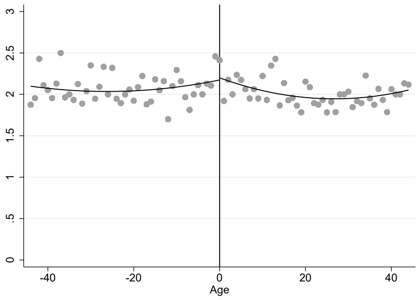

(b) Average net income per consumption unit in € by age in months

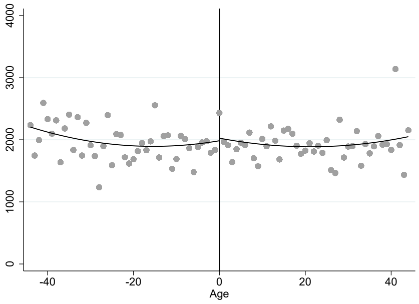

Source: ESPS 2014.

Figure D6. Continuity in the proportion of risk averse individuals - zero cutoff for the 65 years old and bandwidth of 44 months

(a) Percentage of risk averse individuals by age in months

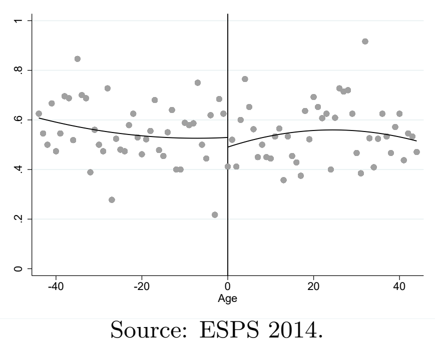

Source: ESPS 2014.

D.1.2 Graphical evidence on the threshold-based sample

Figure D7. Continuity in the socio-demographic characteristics - zero cutoff for the 65 years old and bandwidth of 44 months

(a) Percentage of individuals in a relationship by age in months

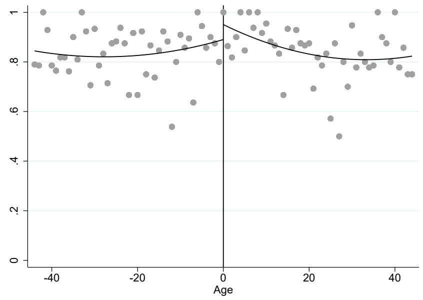

(b) Percentage of males by age in months in months

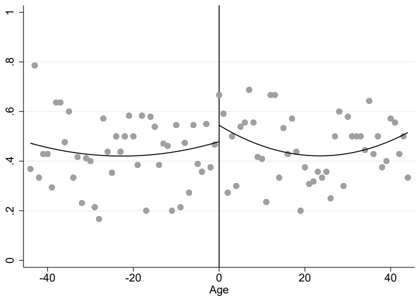

(c) Percentage of individuals with a high school diploma by age in months

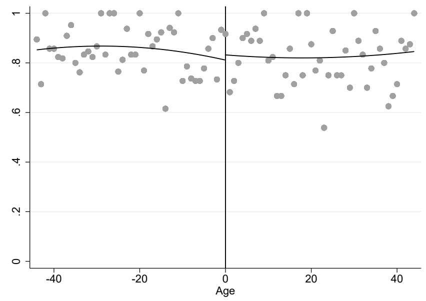

Source: ESPS 2014.

Figure D8. Continuity in the socio-professional categories - zero cutoff for the 65 years old and bandwidth of 44 months

(a) Percentage of Executives by age in months

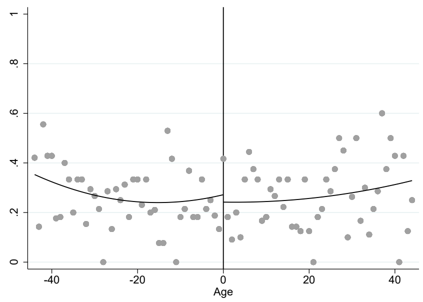

(b) Percentage of Employees by age in months

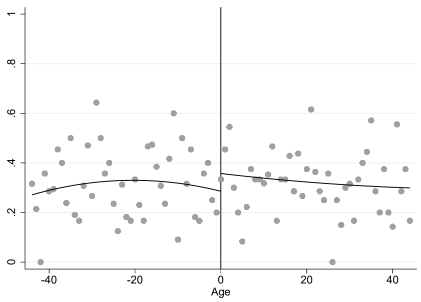

(c) Percentage of Blue Collars by age in months

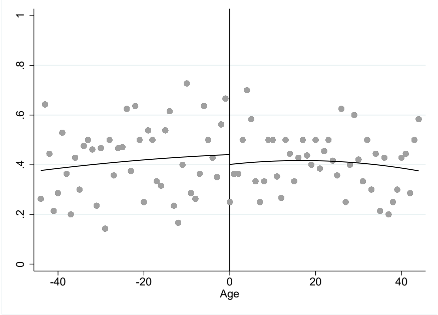

Source: ESPS 2014.

Figure D9. Continuity in the proportion of pensioners - zero cutoff for the 65 years old and bandwidth of 44 months

(a) Percentage of Pensioners by age in months

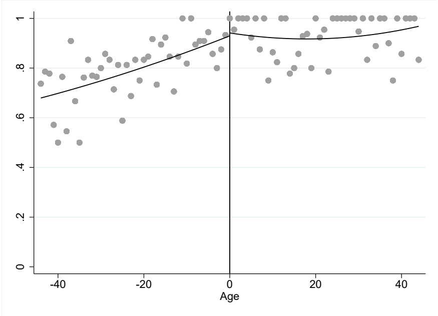

Source: ESPS 2014.

Figure D10. Continuity in household characteristics - zero cutoff for the 65 years old and bandwidth of 44 months

(a) Average number of individuals in the household by age in months

(b) Average net income per consumption unit in € by age in months

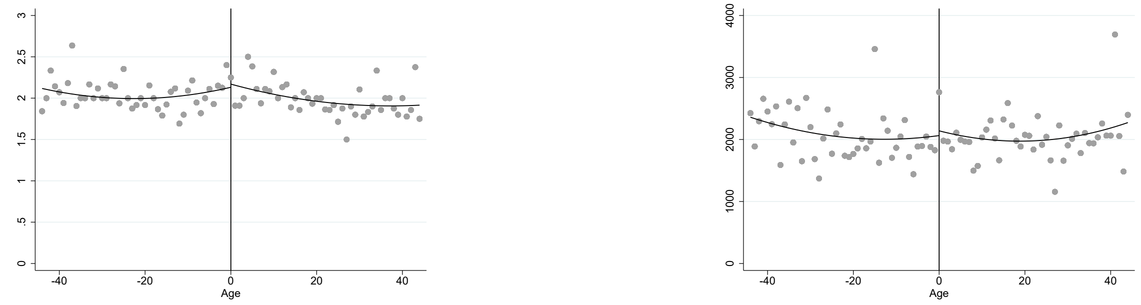

Source: ESPS 2014.

Figure D11. Continuity in the proportion of risk-averse individuals - zero cutoff for the 65 years old and bandwidth of 44 months

(a) Percentage of risk-averse individuals by age in months

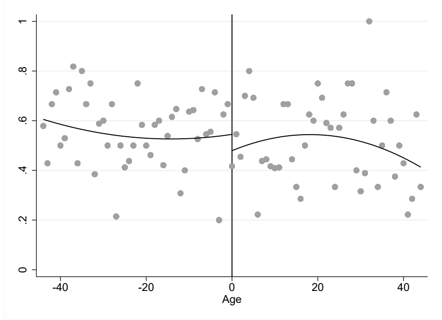

Source: ESPS 2014.

D.1.3 Results of the estimates (non-parametric local linear estimates) on the whole sample

Table D12. Continuity in the characteristics: Non-parametric Local Linear RDD estimates of socio-demographic characteristics, using a bandwidth of 44 months

	In a Relationship	Male	High school Diploma	Chronic Disease	Eligible
	(1)	(2)	(3)	(4)	(5)
$\mathbb{1}_{A_i \geq 65}$	0.04	0.06	-0.00	-0.00	0.01
se	(0.04)	(0.05)	(0.04)	(0.05)	(0.05)
R^2	0.00	0.00	0.00	0.01	0.01
N	1698	1698	1684	1698	1698

Note: Standard errors in parentheses. ***Statistically significant at the 1% level; **Statistically significant at the 5% level; *Statistically significant at the 10% level. Conventional RD estimates with a conventional variance estimator are presented. Source: ESPS 2014.

Table D13. Continuity in the characteristics: Non-parametric Local linear RDD estimates of socio-professional category, using a bandwidth of 44 months

	Executive	Employee	Blue Collar	Pensioner
	(1)	(2)	(3)	(4)
$\mathbb{1}_{A_i \geq 65}$	0.02	0.01	-0.04	0.03
se	(0.04)	(0.05)	(0.05)	(0.03)
R^2	0.00	0.00	0.00	0.00
N	1690	1690	1690	1698

Note: Standard errors in parentheses. ***Statistically significant at the 1% level; **Statistically significant at the 5% level; *Statistically significant at the 10% level. Conventional RD estimates with a conventional variance estimator are presented. Source: ESPS 2014.

Table D14. Continuity in the characteristics: Non-parametric Local Linear RDD estimates of household characteristics, using a bandwidth of 44 months

	Number of individuals	Net income
	(1)	(2)
$\mathbb{1}_{A_i \geq 65}$	0.03	112.77
se	(0.08)	(100.94)
R^2	0.01	0.00
N	1698	1451

Note: Standard errors in parentheses. ***Statistically significant at the 1% level; **Statistically significant at the 5% level; *Statistically significant at the 10% level. Conventional RD estimates with a conventional variance estimator are presented. Source: ESPS 2014.

Table D15. Continuity in the characteristics: Non-parametric Local Linear RDD estimates of preferences, using a bandwidth of 44 months

	Risk averse
	(1)
$\mathbb{1}_{A_i \geq 65}$	0.01
se	(0.04)
R^2	0.00
N	1698

Note: Standard errors in parentheses. ***Statistically significant at the 1% level; **Statistically significant at the 5% level; *Statistically significant at the 10% level. Conventional RD estimates with a conventional variance estimator are presented. Source: ESPS 2014.

D.1.4 Results of the estimates (non parametric local linear estimates) on the threshold-based sample

Table D16. Continuity in the characteristics: Non-parametric Local Linear RDD estimates of socio-demographic characteristics, using a bandwidth of 44 months

	In a Relationship (1)	Male (2)	High school Diploma (3)	Chronic Disease (4)	Eligible (5)
$\mathbb{1}_{A_i \geq 65}$	0.07*	0.07	0.01	-	-
se	(0.04)	(0.06)	(0.05)	-	-
R^2	0.01	0.00	0.00	-	-
N	1130	1130	1120	-	-

Note: Standard errors in parentheses. ***Statistically significant at the 1% level; **Statistically significant at the 5% level; *Statistically significant at the 10% level. Conventional RD estimates with a conventional variance estimator are presented. Source: ESPS 2014.

Table D17. Continuity in the characteristics for the threshold based-sample: Non-parametric Local linear RDD estimates of socio-professional category, using a bandwidth of 44 months

	Executive (1)	Employee (2)	Blue Collar (3)	Pensioner (4)
$\mathbb{1}_{A_i \geq 65}$	0.01	0.06	-0.06	0.01
se	(0.05)	(0.06)	(0.06)	(0.03)
R^2	0.00	0.00	0.00	0.04
N	1125	1125	1125	1130

Note: Standard errors in parentheses. ***Statistically significant at the 1% level; **Statistically significant at the 5% level; *Statistically significant at the 10% level. Conventional RD estimates with a conventional variance estimator are presented. Source: ESPS 2014.

Table D18. Continuity in the characteristics for the threshold-based sample: Non-parametric Local Linear RDD estimates of household characteristics, using a bandwidth of 44 months

	Number of individuals (1)	Net income (2)
$\mathbb{1}_{A_i \geq 65}$	0.07	171.25
se	(0.09)	(127.93)
R^2	0.01	0.00
N	1130	965

Note: Standard errors in parentheses. ***Statistically significant at the 1% level; **Statistically significant at the 5% level; *Statistically significant at the 10% level. Conventional RD estimates with a conventional variance estimator are presented. Source: ESPS 2014.

Table D19. Continuity in the characteristics for the threshold based-sample: Non-parametric Local Linear RDD estimates of preferences, using a bandwidth of 44 months

	Risk averse
	(1)
$\mathbb{1}_{A_i \geq 65}$	0.02
se	(0.05)
R^2	0.00
N	1130

Note: Standard errors in parentheses. ***Statistically significant at the 1% level; **Statistically significant at the 5% level; *Statistically significant at the 10% level. Conventional RD estimates with a conventional variance estimator are presented. Source: ESPS 2014.

D.2 McCrary test for continuity in the number of individuals at the age of 65

Figure D20. Density in the number of individuals per age (0 cut-off for age 65)

(a) McCrary test on the whole sample

(b) McCrary test on the threshold-based sample

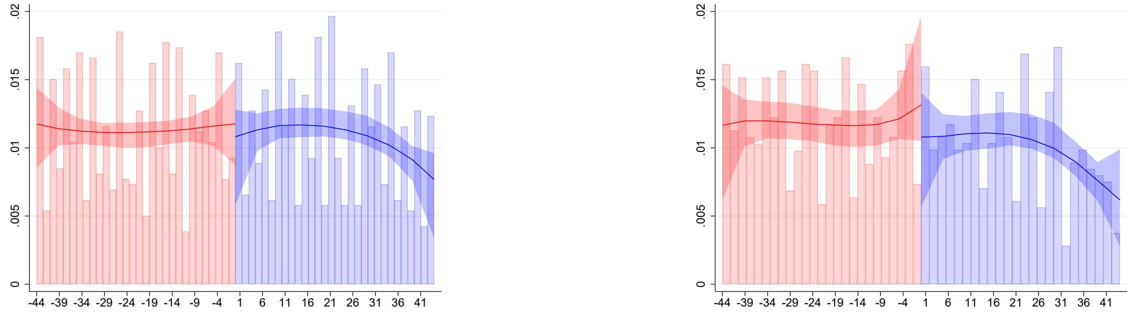

Source: ESPS 2014.

D.3 Retirement and vaccination take-up

Figure D21. Share of pensioners by age

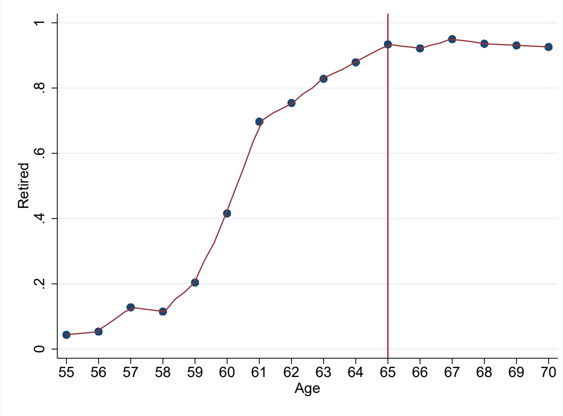

Source: ESPS 2014.

Table D22. Placebo tests: Reduced-form estimates (Equation (4)), using different age thresholds and a bandwidth of 44 month around each threshold

	**Non-parametric Specification** P(Vacc=1)	**Parametric Specification** P(Vacc=1)
$\mathbb{1}_{A_i \geq 60}$	0.03	0.10
se	(0.04)	(0.10)
N	1681	1681
$\mathbb{1}_{A_i \geq 61}$	-0.03	0.01
se	(0.04)	(0.08)
N	1702	1702
$\mathbb{1}_{A_i \geq 62}$	0.01	0.00
se	(0.04)	(0.07)
N	1700	1700
$\mathbb{1}_{A_i \geq 63}$	0.03	0.06
se	(0.04)	(0.05)
N	1723	1723
$\mathbb{1}_{A_i \geq 64}$	-0.04	0.02
se	(0.04)	(0.05)
N	1738	1738

Note: Standard errors in parentheses. ***Statistically significant at the 1% level; **Statistically significant at the 5% level; *Statistically significant at the 10% level. For non-parametric specification, conventional RD estimates with a conventional variance estimator are presented. For the parametric specification, we control for linear trends of age, continuous at the age of X (with X being 60, 61, 62, 63 or 64): $(A_i - X)\mathbb{1}_{A_i \geq X}$ and $(A_i - X)\mathbb{1}_{A_i < X}$. Source: ESPS 2014.

D.4 Placebo tests on individuals with chronic diseases

D.4.1 Graphical evidence

Figure D23. Vaccination outcomes, by age in months, on the sub-sample only composed of individuals with chronic diseases - zero cutoff for the 65 years old and bandwidth of 44 months

(a) Flu vaccination invitation rate, by age in months

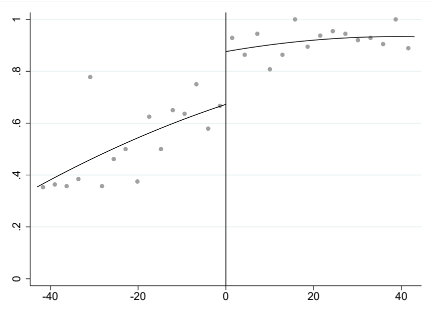

(b) Flu vaccination rate, by age in months

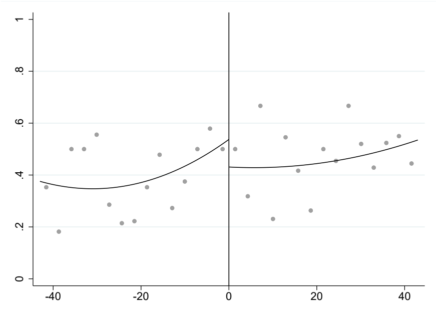

Source: ESPS 2014.

D.4.2 Results of the estimates

Table D24. Non-parametric Local Linear RDD estimates for individuals with chronic diseases, using a bandwidth of 44 months

	Vaccination uptake		
	First Stage	Second Stage	Reduced Form
	(1)	(2)	(3)
$\mathbb{1}_{A_i \geq 65}$	0.20***	-	-0.09
se	(0.08)		(0.09)
Invitation	-	-0.40	-
se		(0.53)	
N	568	568	568

Note: Standard errors in parentheses. ***Statistically significant at the 1% level; **Statistically significant at the 5% level; *Statistically significant at the 10% level. Conventional RD estimates with a conventional variance estimator are presented..
Source: ESPS 2014.

E Main Results

E.1 Non-parametric specification, using different methods for the construction of confidence intervals

Table E1. Regression Discontinuity estimates of equations (2), (3) and (4), using a bandwidth of 44 months around the 65 years old threshold, on the two different samples

	Vaccination Uptake					
	Whole Sample			Threshold-based Sample		
	First Stage (1)	Second Stage (2)	Reduced Form (3)	First Stage (4)	Second Stage (5)	Reduced Form (6)
	**Non-parametric specification: Conventional**					
$\mathbb{1}_{A_i \geq 65}$	0.40***	-	0.06	0.49***	-	0.12**
se	(0.05)		(0.05)	(0.05)		(0.05)
Invitation	-	0.16	-	-	0.25**	-
se		(0.11)			(0.10)	
	**Non-parametric specification: Bias-corrected coeff.**					
$\mathbb{1}_{A_i \geq 65}$	0.38***	-	0.06	0.46***	-	0.13***
se	(0.05)		(0.05)	(0.05)		(0.05)
Invitation	-	0.17	-	-	0.27***	-
se		(0.11)			(0.10)	
	**Non-parametric specification: Bias-corrected coeff.; Robust s.e**					
$\mathbb{1}_{A_i \geq 65}$	0.38***	-	0.06	0.46***	-	0.13**
se	(0.05)		(0.05)	(0.06)		(0.05)
Invitation	-	0.17	-	-	0.27***	-
se		(0.12)			(0.11)	
	**Parametric: Local Linear**					
$\mathbb{1}_{A_i \geq 65}$	0.44***	-	0.06	0.54***	-	0.12***
se	(0.04)		(0.04)	(0.05)		(0.05)
Invitation	-	0.14	-	-	0.22***	-
se		(0.09)			(0.08)	
N	1698	1698	1698	1130	1130	1130

Note: Standard errors in parentheses. ***Statistically significant at the 1% level; **Statistically significant at the 5% level; *Statistically significant at the 10% level. Conventional corresponds to conventional RD estimates with a conventional variance estimator, Bias-corrected to bias-corrected RD estimates with a conventional variance estimator and Robust to bias-corrected RD estimates with a robust variance estimator. For the parametric specification, we control for linear trends of age, continuous at the age of 65: $(A_i - 65)\mathbb{1}_{A_i \geq 65}$ and $(A_i - 65)\mathbb{1}_{A_i < 65}$. Source: ESPS 2014.

E.2 First-stage estimates according to the level of risk aversion

Table E2. Estimates of two first-stage regressions, using a bandwidth of 44 months around the 65 years old threshold, on the two different samples

	Whole Sample		Threshold-based Sample	
	P(R=1)	P(R*RA=1)	P(R=1)	P(R*RA=1)
	**Non-Parametric Specification**			
$\mathbb{1}_{A_i \geq 65}$	0.41***	-0.10***	0.47***	-0.09***
se	(0.05)	(0.02)	(0.06)	(0.02)
$\mathbb{1}_{A_i \geq 65}$*RA	-0.01	0.57***	0.04	0.69***
se	(0.04)	(0.03)	(0.05)	(0.03)
F-test	39.97	316.47	42.24	404.21
	**Parametric Specification**			
$\mathbb{1}_{A_i \geq 65}$	0.44***	-0.11***	0.53***	-0.10***
se	(0.04)	(0.02)	(0.05)	(0.03)
$\mathbb{1}_{A_i \geq 65}$*RA	-0.00	0.63***	0.03	0.75***
se	(0.04)	(0.02)	(0.04)	(0.03)
F-test	61.80	402.80	69.35	499.28
N	1698	1698	1130	1130

Note: We only report the coefficient of interest. Standard errors in parentheses. ***Statistically significant at the 1% level; **Statistically significant at the 5% level; *Statistically significant at the 10% level. For the non-parametric specification, conventional coefficients with conventional RD estimates are presented. For the parametric specification, we control for linear trends of age, continuous at the age of 65: $(A_i - 65)\mathbb{1}_{A_i \geq 65}$ and $(A_i - 65)\mathbb{1}_{A_i < 65}$. Source: ESPS 2014.

E.3 Heterogeneous effects by socio-demographic characteristics

Table E3. Estimates of equation (3), allowing for heterogeneity according to socio-demographic characteristics, using a bandwidth of 44 months around the 65 years old threshold, on the two different samples

	Vaccination Take-up							
	**Marital Status**		**Gender**		**Diploma level**		**Income**	
	Whole Sample	Threshold based Sample	Whole Sample	Threshold based Sample	Whole Sample	Threshold based Sample	Whole Sample	Threshold based Sample
	(1)	(2)	(3)	(4)	(5)	(6)	(7)	(8)
	**Non-Parametric**							
Invit $\times X_i$	0.18	0.09	-0.02	0.02	-0.19*	0.10	-0.06	-0.07
se	(0.11)	(0.11)	(0.09)	(0.09)	(0.11)	(0.10)	(0.09)	(0.09)
Invit	-0.00	0.17	0.17	0.25**	0.33**	0.13	0.25*	0.30***
se	(0.15)	(0.14)	(0.11)	(0.10)	(0.15)	(0.15)	(0.13)	(0.11)
X_i	-0.10	-0.07	0.04	-0.04	0.13**	-0.14**	0.05	0.04
se	(0.08)	(0.06)	(0.06)	(0.05)	(0.07)	(0.06)	(0.06)	(0.05)
	**Parametric**							
Invit $\times X_i$	0.16*	0.04	-0.03	0.01	-0.09	0.07	-0.05	-0.07
se	(0.09)	(0.09)	(0.07)	(0.07)	(0.09)	(0.09)	(0.07)	(0.07)
Invit	-0.00	0.18	0.15*	0.21**	0.23*	0.13	0.20*	0.26***
se	(0.12)	(0.11)	(0.09)	(0.09)	(0.13)	(0.13)	(0.11)	(0.09)
X_i	-0.08	-0.03	0.05	-0.02	0.05	-0.09*	0.04	0.05
se	(0.06)	(0.05)	(0.04)	(0.04)	(0.06)	(0.05)	(0.04)	(0.04)
N	1698	1130	1698	1130	1684	1130	1451	965

Note: Standard errors in parentheses. ***Statistically significant at the 1% level; **Statistically significant at the 5% level; *Statistically significant at the 10% level. For the non-parametric specification, conventional coefficients with conventional RD estimates are presented. For the parametric specification, we control for linear trends of age, continuous at the age of 65: $(A_i - 65)\mathbb{1}_{A_i \geq 65}$ and $(A_i - 65)\mathbb{1}_{A_i < 65}$.
Source: ESPS 2014.

E.4 Profile of compliers, always-takers and never-takers according to several socio-demographic characteristics

Figure E4. Profile of compliers, always takers and never-takers

(a) Percentage of individuals in a relationship by group

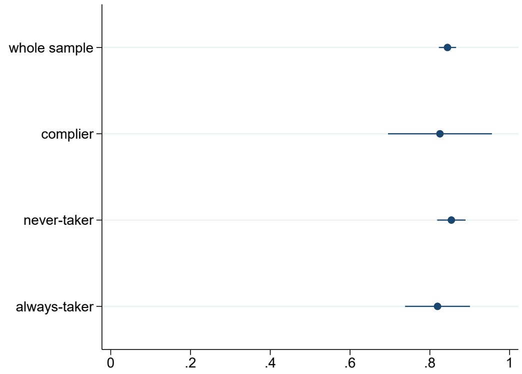

(b) Percentage of males by group

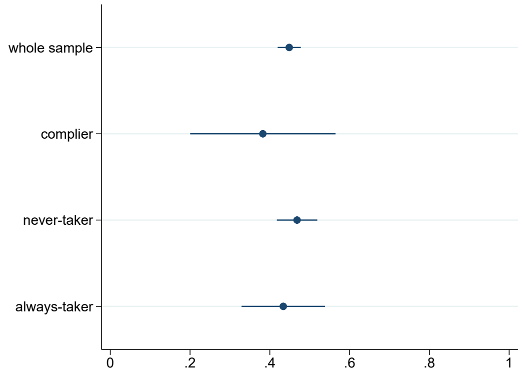

(c) Percentage of individuals with a higher degree by group

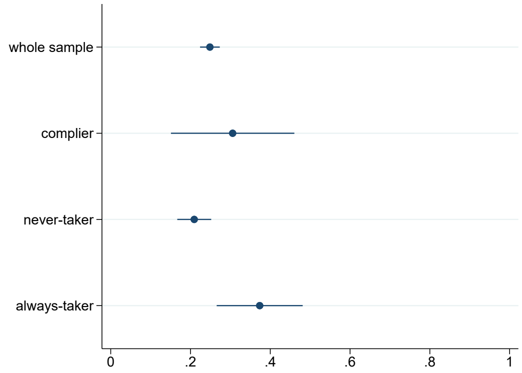

(d) Percentage of individuals with a high income (> median income) by group

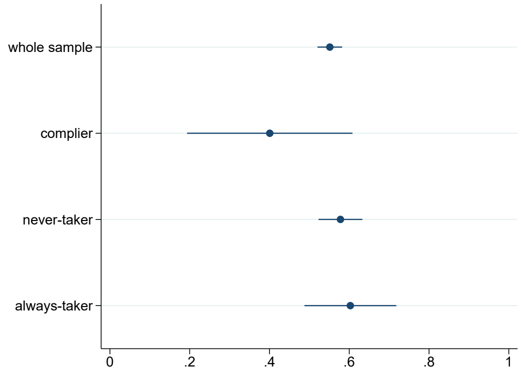

Source: ESPS 2014.

F Robustness checks

F.1 Use of other specifications

Table F1. Regression Discontinuity estimates of equations (2), (3) and (4) using different specifications, with a bandwidth of 44 months around the 65 years old threshold, on the two different samples

	Vaccination uptake					
	**Whole Sample**			**Threshold-based Sample**		
	First Stage (1)	Second Stage (2)	Reduced Form (3)	First Stage (4)	Second Stage (5)	Reduced Form (6)
	**Controls**					
$\mathbb{1}_{A_i \geq 65}$	0.41***	-	0.08	0.41***	-	0.13***
se	(0.05)		(0.05)	(0.08)		(0.05)
Invitation	-	0.18*	-	-	0.26***	-
se		(0.11)			(0.10)	
N	1676	1676	1676	1115	1115	1115
	**Clustered by age - counties**					
$\mathbb{1}_{A_i \geq 65}$	0.40***	-	0.06	0.41***	-	0.12**
se	(0.05)		(0.05)	(0.08)		(0.05)
Invitation	-	0.16	-	-	0.25**	-
se		(0.11)			(0.10)	
N	1698	1698	1698	1130	1130	1130
	**Quadratic**					
$\mathbb{1}_{A_i \geq 65}$	0.33***	-	0.06	0.38***	-	0.13*
se	(0.07)		(0.07)	(0.12)		(0.07)
Invitation	-	0.19	-	-	0.34*	-
se		(0.19)			(0.18)	
N	1698	1698	1698	1130	1130	1130

Note: Standard errors in parentheses. ***Statistically significant at the 1% level; **Statistically significant at the 5% level; *Statistically significant at the 10% level. Conventional RD estimates with a conventional variance estimator. Source: ESPS 2014.

F.2 Use of other bandwidths

Figure F2. Point estimates at the age threshold of 65, for the first stage and the reduced form using different bandwidths

(a) Flu vaccination invitation estimates on the whole sample

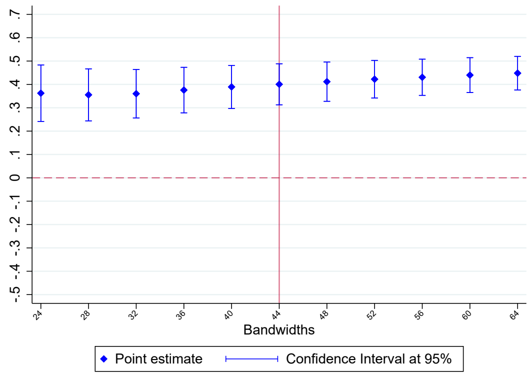

(b) Flu vaccination take-up estimates on the whole sample

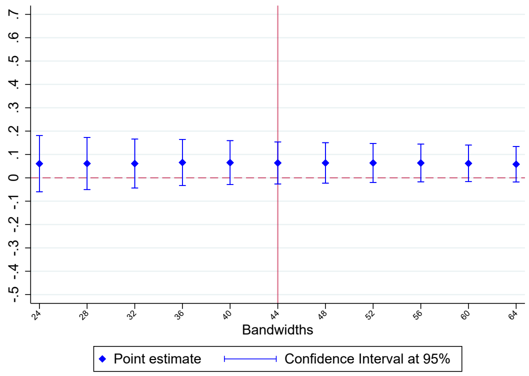

(c) Flu vaccination take-up estimates on the threshold-based sample

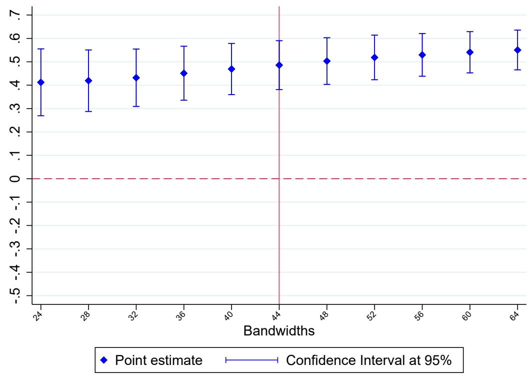

(d) Flu vaccination take-up estimates on the threshold-based sample

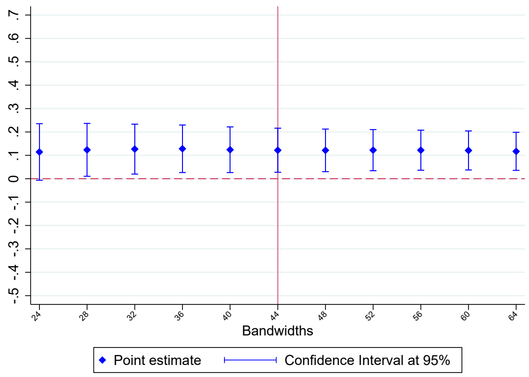

Source: ESPS 2014.

Figure F3. Point estimates at the age threshold of 65 for risk-averse individuals, for the first stage and the reduced form using different bandwidths

(a) Flu vaccination invitation estimates for risk takers on the whole sample

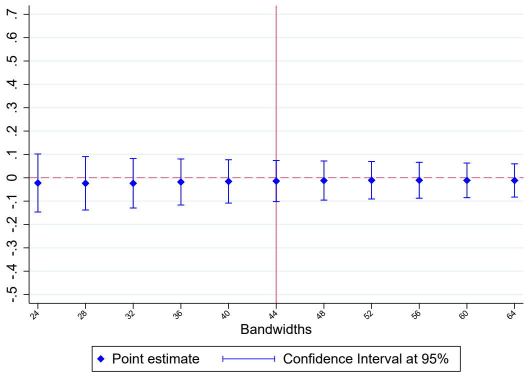

(b) Flu vaccination take-up estimates for risk takers on the whole sample

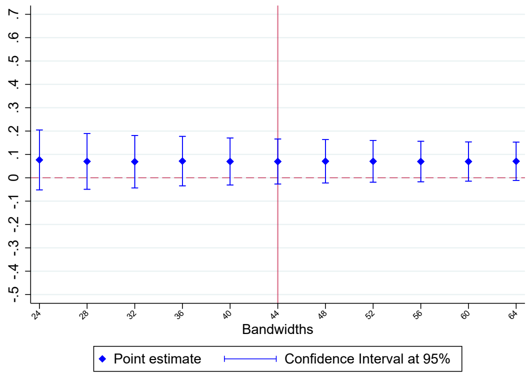

(c) Flu vaccination invitation estimates for risk takers on the threshold-based sample

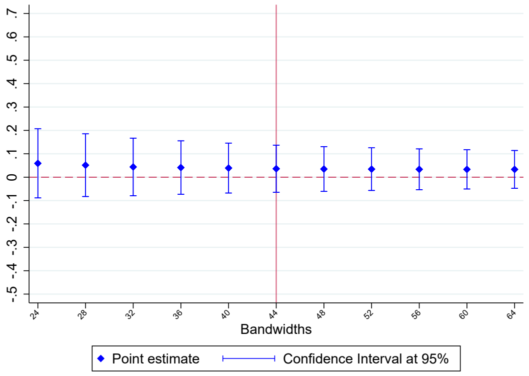

(d) Flu vaccination take-up estimates for risk takers on the threshold-based sample

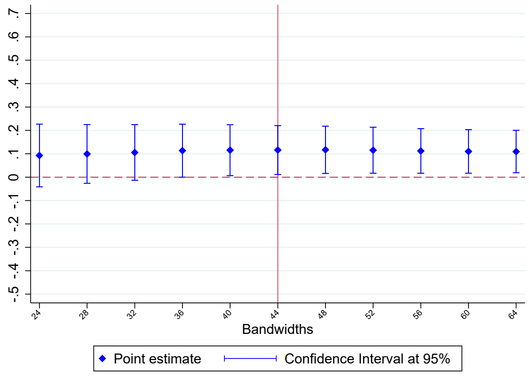

Source: ESPS 2014.

F.3 Placebo tests using different age thresholds

Table F4. Placebo tests: RD estimates of flu vaccination take-up, using a bandwidth of 44 month around the threshold on the whole sample

	**Non-parametric Local Linear**	**Parametric Local Linear**
	(1)	(2)
$\mathbb{1}_{A_i \geq 66}$	0.05	0.06
se	(0.05)	(0.05)
N	1633	1633
$\mathbb{1}_{A_i \geq 67}$	-0.09*	-0.12*
se	(0.05)	(0.07)
N	1578	1578
$\mathbb{1}_{A_i \geq 68}$	-0.03	-0.06
se	(0.06)	(0.10)
N	1470	1470
$\mathbb{1}_{A_i \geq 69}$	0.06	0.18
se	(0.06)	(0.13)
N	1355	1355

Note: Standard errors in parentheses. ***Statistically significant at the 1% level; **Statistically significant at the 5% level; *Statistically significant at the 10% level. For the non-parametric specification, conventional coefficients with conventional RD estimates are presented. For the non-parametric specification, conventional coefficients with conventional RD estimates are presented. For the parametric specification, we control for linear trends of age, continuous at the age of X (with X being 66, 67, 68 or 69): $(A_i - X)\mathbb{1}_{A_i \geq X}$ and $(A_i - X)\mathbb{1}_{A_i < X}$. Source: ESPS 2014.

Table F5. Placebo tests: RD estimates of flu vaccination take-up, using a bandwidth of 44 month around the threshold on the threshold-based sample

	**Non-parametric Specification** (1)	**Parametric Specification** (2)
$\mathbb{1}_{A_i \geq 66}$	0.04	-0.02
se	(0.08)	(0.09)
N	596	596
$\mathbb{1}_{A_i \geq 67}$	0.00	-0.09
se	(0.08)	(0.11)
N	619	619
$\mathbb{1}_{A_i \geq 68}$	0.00	-0.04
se	(0.09)	(0.15)
N	586	586
$\mathbb{1}_{A_i \geq 69}$	0.05	0.26
se	(0.09)	(0.18)
N	573	573

Note: Standard errors in parentheses. ***Statistically significant at the 1% level; **Statistically significant at the 5% level; *Statistically significant at the 10% level. For the non-parametric specification, conventional coefficients with conventional RD estimates are presented. For the parametric specification, we control for linear trends of age, continuous at the age of X (with X being 66, 67, 68 or 69): $(A_i - X)\mathbb{1}_{A_i \geq X}$ and $(A_i - X)\mathbb{1}_{A_i < X}$. Source: ESPS 2014.

F.4 Alternative measure of risk aversion

Table F6. Regression Discontinuity estimates of equation (5), using a bandwidth of 44 months around the 65 years old threshold, on the two different samples – Heterogeneity according to the alternative definition of risk aversion (risk averse individuals: those who answer 5 or less to the question on risk aversion)

	**Vaccination uptake**	
	Whole Sample (1)	Threshold-based Sample (2)
	**Non-Parametric**	
Invitation	0.01	0.11
se	(0.14)	(0.12)
Invitation × Risk averse	0.19*	0.18**
se	(0.10)	(0.09)
Risk Averse	-0.05	-0.02
se	(0.07)	(0.05)
	**Parametric**	
Invitation	0.01	0.10
se	(0.12)	(0.10)
Invitation × Risk Averse	0.18**	0.15*
se	(0.09)	(0.08)
Risk Averse	-0.05	-0.02
se	(0.06)	(0.05)
N	1698	1130

Note: Standard errors in parentheses. ***Statistically significant at the 1% level; **Statistically significant at the 5% level; *Statistically significant at the 10% level. For the non-parametric specification, conventional RD estimates with a conventional variance estimator are presented. For the parametric specification, we control for linear trends of age, continuous at the age of 65: $(A_i - 65)\mathbb{1}_{A_i \geq 65}$ and $(A_i - 65)\mathbb{1}_{A_i < 65}$
Source: ESPS 2014.

F.5 Restricted samples

F.5.1 Sample excluding all individuals born between October and December 1949

Table F7. Regression Discontinuity estimates of equations (2), (3) and (4), using a bandwidth of 44 months around the 65 years old threshold, on the two different samples

	Vaccination uptake					
	**Whole Sample**			**Threshold-based Sample**		
	First Stage	Second Stage	Reduced Form	First Stage	Second Stage	Reduced Form
	(1)	(2)	(3)	(4)	(5)	(6)
	**Non-parametric: Conventional**					
$\mathbb{1}_{A_i \geq 65}$	0.42***	-	0.04	0.54***	-	0.10*
se	(0.05)		(0.05)	(0.06)		(0.05)
Invitation	-	0.10	-	-	0.19*	-
se		(0.12)			(0.10)	
	**Non-parametric: Bias-corrected**					
$\mathbb{1}_{A_i \geq 65}$	0.40***	-	0.04	0.52***	-	0.11**
se	(0.05)	-	(0.05)	(0.06)	-	(0.05)
Invitation	-	0.11	-	-	0.21**	-
se		(0.12)			(0.11)	
	**Non-parametric: Robust**					
$\mathbb{1}_{A_i \geq 65}$	0.40***	-	0.04	0.52***	-	0.11*
se	(0.05)		(0.05)	(0.06)		(0.06)
Invitation	-	0.11	-	-	0.21*	-
se		(0.13)			(0.11)	
	**Parametric: Local Linear**					
$\mathbb{1}_{A_i \geq 65}$	0.47***	-	0.05	0.60***	-	0.10*
se	(0.04)		(0.05)	(0.05)		(0.05)
Invitation	-	0.10	-	-	0.16*	-
se		(0.12)			(0.08)	
N	1632	1632	1632	1079	1079	1079

Note: Standard errors in parentheses. ***Statistically significant at the 1% level; **Statistically significant at the 5% level; *Statistically significant at the 10% level. Conventional corresponds to conventional RD estimates with a conventional variance estimator, Bias-corrected to bias-corrected RD estimates with a conventional variance estimator and Robust to bias-corrected RD estimates with a robust variance estimator. For local linear estimates, we control for linear trends of age, continuous at the age of 65: $(A_i - 65)\mathbb{1}_{A_i \geq 65}$ and $(A_i - 65)\mathbb{1}_{A_i < 65}$. Source: ESPS 2014.

Table F8. Regression Discontinuity estimates of equation (5), using a bandwidth of 44 months around the 65 years old threshold, on the two different samples – Heterogeneity according to risk aversion.

	**Vaccination uptake**	
	Whole Sample (1)	Threshold-based Sample (2)
	**Non-Parametric**	
Invitation	0.05	0.11
se	(0.12)	(0.10)
Invitation × Risk Averse	0.13	0.18**
se	(0.08)	(0.08)
Risk Averse	-0.03	-0.01
se	(0.05)	(0.04)
	**Parametric**	
Invitation	0.04	0.08
se	(0.10)	(0.09)
Invitation × Risk Averse	0.12*	0.16**
se	(0.07)	(0.07)
Risk Averse	-0.03	-0.01
se	(0.04)	(0.04)
N	1632	1079

Note: Standard errors in parentheses. ***Statistically significant at the 1% level; **Statistically significant at the 5% level; *Statistically significant at the 10% level. For the non-parametric specification, conventional RD estimates with a conventional variance estimator are presented. For the parametric specification, we control for linear trends of age, continuous at the age of 65: $(A_i - 65)\mathbb{1}_{A_i \geq 65}$ and $(A_i - 65)\mathbb{1}_{A_i < 65}$. Source: ESPS 2014.

F.5.2 Sample excluding all individuals surveyed during the vaccination campaign (from October 2014 onwards)

Table F9. Regression Discontinuity estimates of equations (2), (3) and (4), using a bandwidth of 44 months around the 65 years old threshold, on the two different samples

	**Vaccination uptake**					
	**Whole Sample**			**Threshold-based Sample**		
	First Stage	Second Stage	Reduced Form	First Stage	Second Stage	Reduced Form
	(1)	(2)	(3)	(4)	(5)	(6)
	**Non-parametric: Conventional**					
$\mathbb{1}_{A_i \geq 65}$	0.55***	-	0.04	0.70***	-	0.13**
se	(0.05)	-	(0.06)	(0.06)	-	(0.06)
Invitation	-	0.08	-	-	0.18**	-
se	-	(0.10)			(0.08)	
	**Non-parametric: Bias-corrected**					
$\mathbb{1}_{A_i \geq 65}$	0.54***	-	0.04	0.69***	-	0.13**
se	(0.05)		(0.06)	(0.06)		(0.06)
Invitation	-	0.08	-	-	0.19**	-
se	-	(0.10)			(0.08)	
	**Non-parametric: Robust**					
$\mathbb{1}_{A_i \geq 65}$	0.54***	-	0.04	0.69***	-	0.13**
se	(0.06)		(0.06)	(0.06)		(0.06)
Invitation	-	0.08	-	-	0.19**	-
se	-	(0.11)			(0.09)	
	**Parametric: Local Linear**					
$\mathbb{1}_{A_i \geq 65}$	0.56***	-	0.04	0.71***	-	0.12**
se	(0.05)		(0.05)	(0.05)		(0.06)
Invitation	-	0.08	-	-	0.17**	-
se	-	(0.10)			(0.08)	
N	1041	1041	1041	679	679	679

Note: Standard errors in parentheses. ***Statistically significant at the 1% level; **Statistically significant at the 5% level; *Statistically significant at the 10% level. For the non-parametric specification, conventional RD estimates with a conventional variance estimator are presented. For the parametric specification, we control for linear trends of age, continuous at the age of 65: $(A_i - 65)\mathbb{1}_{A_i \geq 65}$ and $(A_i - 65)\mathbb{1}_{A_i < 65}$. Source: ESPS 2014.

Table F10. Regression Discontinuity estimates of equation (5), using a bandwidth of 44 months around the 65 years old threshold, on the two different samples – Heterogeneity according to risk aversion.

	**Vaccination uptake**	
	Whole Sample	Threshold-based Sample
	(1)	(2)
	**Non-Parametric**	
Invitation	-0.01	0.08
se	(0.11)	(0.08)
Invitation × Risk Averse	0.18*	0.21**
se	(0.10)	(0.08)
Risk Averse	-0.01	0.04
se	(0.06)	(0.05)
	**Parametric**	
Invitation	0.01	0.09
se	(0.10)	(0.08)
Invitation × Risk Averse	0.14*	0.16**
se	(0.08)	(0.08)
Risk Averse	-0.00	0.04
se	(0.05)	(0.04)
N	1041	679

Note: Standard errors in parentheses. ***Statistically significant at the 1% level; **Statistically significant at the 5% level; *Statistically significant at the 10% level. For the non-parametric specification, conventional RD estimates with a conventional variance estimator are presented. For the parametric specification, we control for linear trends of age, continuous at the age of 65: $(A_i - 65)\mathbb{1}_{A_i \geq 65}$ and $(A_i - 65)\mathbb{1}_{A_i < 65}$. Source: ESPS 2014.

F.5.3 Sample restricted to individuals from households where all members fall on the same side of the threshold

Table F11. Regression Discontinuity estimates of equations (2), (3) and (4), using a bandwidth of 44 months around the 65 years old threshold, on the two different samples

	**Vaccination uptake**					
	**Whole Sample**			**Threshold-based Sample**		
	First Stage (1)	Second Stage (2)	Reduced Form (3)	First Stage (4)	Second Stage (5)	Reduced Form (6)
	**Non-parametric: Conventional**					
$\mathbb{1}_{A_i \geq 65}$	0.40***	-	0.05	0.46***	-	0.12*
se	(0.05)	-	(0.06)	(0.07)	-	(0.06)
Invitation	-	0.12	-	-	0.26*	-
se	-	(0.14)			(0.14)	
	**Non-parametric: Bias-corrected**					
$\mathbb{1}_{A_i \geq 65}$	0.37***	-	0.05	0.44***	-	0.12*
se	(0.05)		(0.06)	(0.07)		(0.06)
Invitation	-	0.12	-	-	0.28**	-
se	-	(0.14)			(0.14)	
	**Non-parametric: Robust**					
$\mathbb{1}_{A_i \geq 65}$	0.37***	-	0.05	0.44***	-	0.12*
se	(0.06)		(0.06)	(0.07)		(0.07)
Invitation	-	0.12	-	-	0.28*	-
se	-	(0.15)			(0.15)	
	**Parametric: Local Linear**					
$\mathbb{1}_{A_i \geq 65}$	0.45***	-	0.04	0.54***	-	0.11*
se	(0.05)		(0.05)	(0.06)		(0.06)
Invitation	-	0.08	-	-	0.20*	-
se	-	(0.12)			(0.11)	
N	1222	1222	1222	820	820	820

Note: Standard errors in parentheses. ***Statistically significant at the 1% level; **Statistically significant at the 5% level; *Statistically significant at the 10% level. Conventional corresponds to conventional RD estimates with a conventional variance estimator, Bias-corrected to bias-corrected RD estimates with a conventional variance estimator and Robust to bias-corrected RD estimates with a robust variance estimator. For the parametric specification, we control for linear trends of age, continuous at the age of 65: $(A_i - 65)\mathbb{1}_{A_i \geq 65}$ and $(A_i - 65)\mathbb{1}_{A_i < 65}$. Source: ESPS 2014.

Table F12. Regression Discontinuity estimates of equation (5), using a bandwidth of 44 months around the 65 years old threshold, on the two different samples – Heterogeneity according to risk aversion.

	**Vaccination uptake**	
	Whole Sample	Threshold-based Sample
	(1)	(2)
	**Non-Parametric**	
Invitation	0.04	0.15
se	(0.14)	(0.14)
Invitation × Risk Averse	0.17*	0.23**
se	(0.10)	(0.09)
Risk Averse	-0.04	-0.03
se	(0.06)	(0.05)
	**Parametric**	
Invitation	0.02	0.12
se	(0.12)	(0.11)
Invitation × Risk Averse	0.12	0.16**
se	(0.08)	(0.08)
Risk Averse	-0.02	-0.02
se	(0.05)	(0.04)
N	1222	817

Note: Standard errors in parentheses. ***Statistically significant at the 1% level; **Statistically significant at the 5% level; *Statistically significant at the 10% level. For the non-parametric specification, conventional RD estimates with a conventional variance estimator are presented. For the parametric specification, we control for linear trends of age, continuous at the age of 11: $(A_i - 65)\mathbb{1}_{A_i \geq 65}$ and $(A_i - 65)\mathbb{1}_{A_i < 65}$. Source: ESPS 2014.

G Theoretical framework

The individual has 2 possible actions: getting vaccinated against the flu or not. The expected utility of being vaccinated is :

$$EU(V) = p_s u(-c_s) + (1 - p_s)u(0) \tag{1}$$

The expected utility of not being vaccinated is :

$$EU(NV) = p_i u(-c_i) + (1 - p_i)u(0) \tag{2}$$

An individual opts for vaccination if and only if :

$$\begin{aligned} EU(V) \geq EU(NV) &\iff p_s u(-c_s) + (1 - p_s)u(0) \geq p_i u(-c_i) + (1 - p_i)u(0) \\ &\iff p_s(-e^{\beta c_s}) + (1 - p_s)(-e^0) \geq p_i(-e^{\beta c_i}) + (1 - p_i)(-e^0) \\ &\iff -p_s e^{\beta c_s} - (1 - p_s) \geq -p_i e^{\beta c_i} - (1 - p_i) \\ &\iff -p_s e^{\beta c_s} - 1 + p_s \geq -p_i e^{\beta c_i} - 1 + p_i \\ &\iff \underbrace{-p_s e^{\beta c_s} + p_i e^{\beta c_i}}_{f(\beta)} \geq p_i - p_s \end{aligned} \tag{3}$$

Denoting $f(\beta) = -p_s e^{\beta c_s} + p_i e^{\beta c_i}$, an individual gets vaccinated if $f(\beta) \geq p_i - p_s$ and does not get vaccinated if $f(\beta) < p_i - p_s$.
Let $\tilde{\beta}$ be the solution of $f(\tilde{\beta}) = p_i - p_s$

Proposition 1 *Before receiving the letter, the individuals' optimal choice of vaccination is:*

	$c_s - c_i > 0$	$c_s - c_i < 0$
$\frac{p_i c_i}{p_s c_s} < 1$	(1) NV $\forall\beta$	(3) V $\Leftrightarrow \beta > \tilde{\beta}$
$\frac{p_i c_i}{p_s c_s} > 1$	(2) V $\Leftrightarrow \beta < \tilde{\beta}$	(4) V $\forall\beta$

Proof. $lim_{\beta\to 0} f(\beta) = lim_{\beta\to 0}(-p_s e^0 + p_i e^0) = p_i - p_s$
If $c_s > c_i$ then $lim_{\beta\to\infty} f(\beta) = -\infty$
If $c_s < c_i$ then $lim_{\beta\to\infty} f(\beta) = \infty$
Let's derive f and study its sign.

$$f'(\beta) = -p_s c_s e^{\beta c_s} + p_i c_i e^{\beta c_i} \tag{4}$$

$$\begin{aligned} f'(\beta) > 0 &\iff -p_s c_s e^{\beta c_s} + p_i c_i e^{\beta c_i} > 0 \\ &\iff -p_s c_s \frac{e^{\beta c_s}}{e^{\beta c_s}} + p_i c_i \frac{e^{\beta c_i}}{e^{\beta c_s}} > 0 \\ &\iff -p_s c_s + p_i c_i e^{\beta(c_i - c_s)} > 0 \\ &\iff ln(p_i c_i e^{\beta(c_i - c_s)}) > ln(p_s c_s) \\ &\iff ln(p_i c_i) + ln(e^{\beta(c_i - c_s)}) > ln(p_s c_s) \\ &\iff ln\frac{p_i c_i}{p_s c_s} > \beta(c_s - c_i) \end{aligned} \tag{5}$$

- If $c_s - c_i > 0$ then $f'(\beta) > 0 \Leftrightarrow ln\frac{p_i c_i}{p_s c_s} > \beta(c_s - c_i) \Leftrightarrow \frac{1}{(c_s - c_i)} ln\frac{p_i c_i}{p_s c_s} \equiv \beta^* > \beta$
  - If $\frac{p_i c_i}{p_s c_s} < 1$ then $\beta^* < 0$ and $f'(\beta) < 0$ for all β. As $f(0) = p_i - p_s$ then $f(\beta) < p_i - p_s$ and thus the individual decides not to be vaccinated (see Equation 3)
  - If $\frac{p_i c_i}{p_s c_s} > 1$ then $\beta^* > 0$ and $f'(\beta) > 0$ if and only if $\beta^* \geq \beta$. As $lim_{\beta\to\infty} f(\beta) = -\infty$, there exist a unique $\tilde{\beta} > 0$ such that $f(\beta) = p_i - p_s$. Thus $f(\beta) > p_i - p_s$ if and only if $\beta < \tilde{\beta}$. Given Equation 3, the individual gets vaccinated if and only if $\beta < \tilde{\beta}$
- If $c_s - c_i < 0$ then $f'(\beta) > 0 \frac{1}{(c_s - c_i)} ln\frac{p_i c_i}{p_s c_s} \equiv \beta^* < \beta$
  - If $\frac{p_i c_i}{p_s c_s} > 1$ then $\beta^* < 0$ and $f'(\beta) > 0$ for all β. As $f(0) = p_i - p_s$ then $f(\beta) > p_i - p_s$ and thus the individual decides to get vaccinated whatever the value of β (see Equation 3)
  - If $\frac{p_i c_i}{p_s c_s} < 1$ then $\beta^* > 0$ and $f'(\beta) < 0$ if and only if $\beta^* \geq \beta$. As $lim_{\beta\to 0} f(\beta) = \infty$, there exists a unique $\tilde{\beta} > 0$ such that $f(\beta) = p_i - p_s$. Thus $f(\beta) < p_i - p_s$ if and only if $\beta < \tilde{\beta}$. The individual does not get vaccinated if and only if $\beta < \tilde{\beta}$.

How does the receipt of the letter change individuals' behavior? Let's concentrate on cases 2 and 3, as cases 1 and 4 are unrealistic.

By informing individuals about the risks of the disease, the leaflet changes individuals' initial values of c_i and p_i, and the voucher included in it changes the value of c_s.However, the invitation letter does not modify p_s Lets denote (c_i, p_i) as the cost of contracting the disease and the probability of contracting it before receiving the letter and (c_i', p_i') those after the receipt of the letter, such that $c_i' \geq c_i$ and $p_i' \geq p_i$. Similarly, let us denote (c_s, p_s) as the cost of the vaccine and the probability of complications due to the injection and (c_s', p_s) those after the receipt of the letter, such that $c_s' < c_s$.

Let g be a function such that $g(\beta, c_i', p_i', c_s') = -p_s e^{\beta c_s'} + p_i' e^{\beta c_i'} + p_s - p_i'$. The individual decides to be vaccinated if and only if $g(\beta, c_i', p_i', c_s') \geq 0$.

First, we show that g is increasing in c_i', p_i' and c_s'.

$$\begin{aligned} g(\beta, c_i', p_i', c_s') - g(\beta, c_i, p_i, c_s) &= -p_s e^{\beta c_s'} + p_i' e^{\beta c_i'} + p_s - p_i' + p_s e^{\beta c_s} - p_i e^{\beta c_i} - p_s + p_i \\ &= p_i' e^{\beta c_i'} - p_i' - p_i e^{\beta c_i} + p_i + p_s(e^{\beta c_s} - e^{\beta c_s'}) \\ &= p_i'(e^{\beta c_i'} - 1) - p_i(e^{\beta c_i} - 1) + p_s(e^{\beta c_s} - e^{\beta c_s'}) \\ &\geq p_i(e^{\beta c_i'} - 1) - p_i(e^{\beta c_i} - 1) + p_s(e^{\beta c_s} - e^{\beta c_s'}) \\ &\text{As } p_i(e^{\beta c_i'} - 1) - p_i(e^{\beta c_i} - 1) = p_i(e^{\beta c_i'} - e^{\beta c_i}) \geq 0 \\ &\text{And } p_s(e^{\beta c_s} - e^{\beta c_s'}) \geq 0 \end{aligned} \tag{6}$$

In case of $\frac{p_i c_i}{p_s c_s} < 1$ and $c_s - c_i < 0$ (case 2), $c_s' - c_i'$ remains negative because $c_i' \geq c_i$ and $c_s' < c_s$.

- If $\frac{p_i' c_i'}{p_s c_s'}$ becomes greater than 1, then the agent vaccinates whatever β.
- If $\frac{p_i' c_i'}{p_s c_s'}$ remains below 1, then the agent does not vaccinate if $\beta < \tilde{\beta}$. Let us now define $\tilde{\beta}'$ as solution of $f(\beta) = p_i' - p_s$. As g increases with (c_i', p_i', c_s'), we get: $g(\tilde{\beta}', c_i', p_i', c_s') \geq g(\tilde{\beta}', c_i, p_i, c_s) = 0$. By definition of $\tilde{\beta}'$, $g(\beta', c_i', p_i', c_s') < 0$ for all $\beta' < \tilde{\beta}'$. Therefore, $\tilde{\beta}' \leq \tilde{\beta}$. As a result, agents with $\beta \in [\tilde{\beta}'; \tilde{\beta}]$ do not get vaccinated if they don't receive the invitation letter but get vaccinated if they do.

In case of $\frac{p_i c_i}{p_s c_s} > 1$ and $c_s - c_i > 0$ (case 3), $\frac{p_i' c_i'}{p_s c_s'}$ remains greater than 1 as $c_i' \geq c_i$ and $p_i' \geq p_i$ and $c_s' < c_s$.

- If $c_s' - c_i'$ becomes less than 0, then the agent vaccinates whatever β
- If $c_s' - c_i'$ remains below 0, the agent does not vaccinate if $\beta > \tilde{\beta}$. Similarly as in case 2, we have $\tilde{\beta}$ solution of $f(\beta) = p_i' - p_s$. By definition of $\tilde{\beta}'$, $g(\beta', c_i', p_i', c_s') < 0$ for all $\beta' > \tilde{\beta}'$. Therefore, $\tilde{\beta}' \geq \tilde{\beta}$. As a result, agents with $\beta \in [\tilde{\beta}; \tilde{\beta}']$ do not get vaccinated if they don't receive the invitation letter but get vaccinated if they do.
